# The Current Status of Research on Gibberellin Biosynthesis

**DOI:** 10.1093/pcp/pcaa092

**Published:** 2020-07-11

**Authors:** Peter Hedden

**Affiliations:** 1 Laboratory of Growth Regulators, Palack� University & Institute of Experimental Botany of the Czech Academy of Sciences, Šlechtitelů 27, 78371 Olomouc, Czech Republic; 2 Rothamsted Research, West Common, Harpenden, Hertfordshire AL5 2JQ, UK

**Keywords:** Gibberellin metabolism

## Abstract

Gibberellins are produced by all vascular plants and several fungal and bacterial species that associate with plants as pathogens or symbionts. In the 60 years since the first experiments on the biosynthesis of gibberellic acid in the fungus *Fusarium fujikuroi*, research on gibberellin biosynthesis has advanced to provide detailed information on the pathways, biosynthetic enzymes and their genes in all three kingdoms, in which the production of the hormones evolved independently. Gibberellins function as hormones in plants, affecting growth and differentiation in organs in which their concentration is very tightly regulated. Current research in plants is focused particularly on the regulation of gibberellin biosynthesis and inactivation by developmental and environmental cues, and there is now considerable information on the molecular mechanisms involved in these processes. There have also been recent advances in understanding gibberellin transport and distribution and their relevance to plant development. This review describes our current understanding of gibberellin metabolism and its regulation, highlighting the more recent advances in this field.

## Introduction

The name gibberellin encompasses a large group of diterpenoid carboxylic acids that are classified as such according to their structure. They were first discovered as metabolites of the fungus *Gibberella fujikuroi*, reclassified as *Fusarium fujikuro*i, that promoted growth in higher plants, and their suspected presence in plants as natural hormones was confirmed in the late-1950s (MacMillan and Suter 1958). The fungal gibberellins were given the trivial names gibberellin A_1_, A_2_, etc. (Takahashi et�al. 1955), a system that was adopted for gibberellins from all sources, with numbers being assigned in order of discovery and structural characterization (MacMillan and Takahashi 1968). Currently, 136 gibberellins have been assigned numbers, but the last to be characterized was over 15 years ago and it is unlikely that this system of nomenclature will be continued. The most abundant gibberellin present in *F. fujikuroi* and the first to be structurally characterized is gibberellin A_3_, which is also known as gibberellic acid (Curtis and Cross 1954). This gibberellin is produced on an industrial scale in fungal cultures for application in agriculture, the largest use being in the production of seedless grapes (Rademacher 2016). However, although it is present in some higher plant species as a minor gibberellin, there is little evidence that gibberellic acid plays an important role in plants.

It is now common practice to abbreviate gibberellin A_*x*_ as GA_*x*_, with the generic abbreviation GA used for gibberellin. This has resulted in some confusion, with many workers assuming that GA is an abbreviation for gibberellic acid, i.e. GA_3_. The name gibberellin A was used in early gibberellin research on the fungal metabolites to distinguish it from a second biologically active fraction, which was named gibberellin B, although the identity of this material is still unclear (Yabuta and Sumiki 1938). An unfortunate consequence is that gibberellic acid has become synonymous with gibberellin and its concentration rather than that of the more relevant biologically active compounds GA_1_ and GA_4_ is frequently measured in plant tissues. GA_3_ differs from GA_1_ in possessing a double bond between C-atoms 1 and 2 (see Fig.�1), which protects it from 2β-hydroxylation, a major mechanism for inactivating GAs in higher plants (see below). An inability to regulate the concentration of GA_3_ by this mechanism may explain its absence or low levels in plant organs. In contrast, its production by the phytopathogenic *F. fujikuroi* would benefit the fungus by compromising the plant host’s ability to protect itself from a high GA dosage.

**Fig 1:**
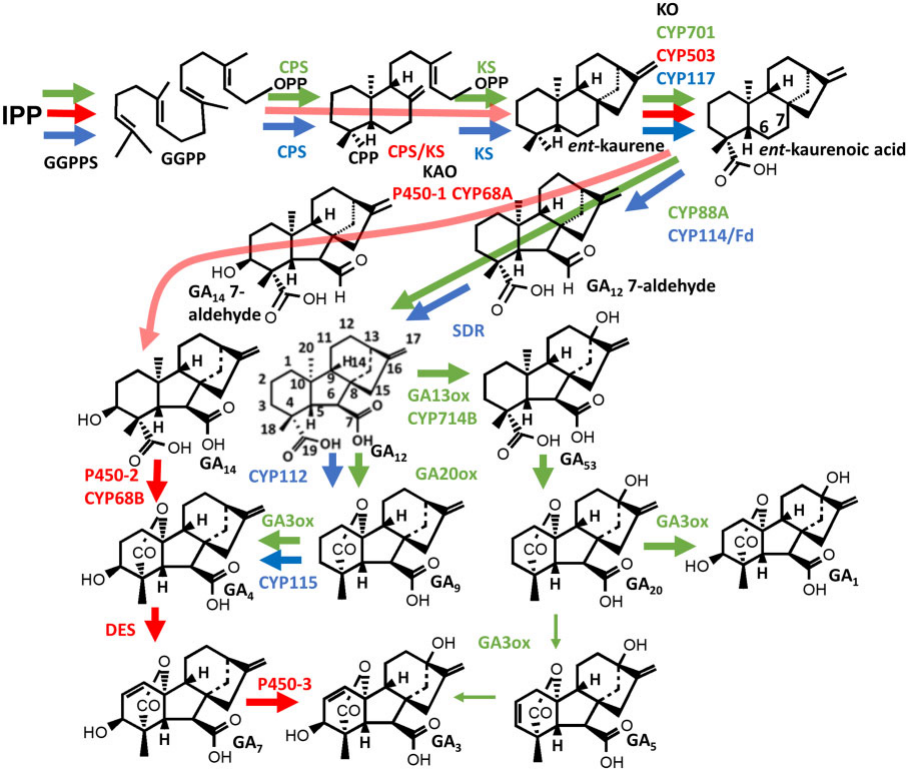
An overview of GA biosynthesis, comparing pathways for higher plants (in green), the fungus *F. fujikuroi* (red) and bacteria (blue) to biological active products. Enzymes are indicated in the respective colors. C-atom numbers are given for the C_20_-GA, GA_12_. GA13ox, GA 13-oxidase; SDR, short-chain dehydrogenase/reductase.

Gibberellins are thought to be present in all vascular plants: in lower plants, such as lycophytes and ferns, GAs are involved in reproductive development (Tanaka et�al. 2014, Miyazaki et�al. 2018), whereas in higher plants GA function has expanded to the promotion of organ growth through enhanced cell elongation and/or cell division and, in many species, activation of developmental processes, such as seed germination, maturation and induction of flowering (Sponsel 2016). Gibberellins are also produced by some fungal and bacterial species that associate with plants, either as pathogens or symbionts. In these cases, GAs appear to have no developmental function in the producing organism but act on the plant host to aid infection by suppressing immunity (Navarro et�al. 2008, Wiemann et�al. 2013, Lu et�al. 2015, Pieterse et�al. 2014) or, in the case of nitrogen-fixing rhizobium bacteria, to regulate nodule formation (Tatsukami and Ueda 2016, McAdam et�al. 2018). Remarkably, within the three kingdoms, plants, fungi and bacteria, the ability to synthesize these complex molecules has been acquired independently by convergent evolution (Nett et�al. 2017). Furthermore, the ability to inactivate GAs by 2β-hydroxylation is present only in higher plants: gymnosperms and angiosperms. Our knowledge of GA biosynthesis and signal transduction has progressed rapidly in recent years, enabled particularly by advances in molecular genetics and the utilization of mutants, either naturally occurring or created through forward or reverse genetic approaches. This review describes our current understanding, including the more recent developments. A historical account can be found in Hedden and Sponsel (2015). Fig.�1 provides an overview of GA biosynthesis in the three kingdoms, plants, fungi and bacteria, highlighting the distinct enzymes utilized for the same or similar reactions, while a more detailed biosynthetic pathway in plants is presented in Figs.�2, 3. Fig.�1 also includes the C-atom numbering system.

**Fig 2:**
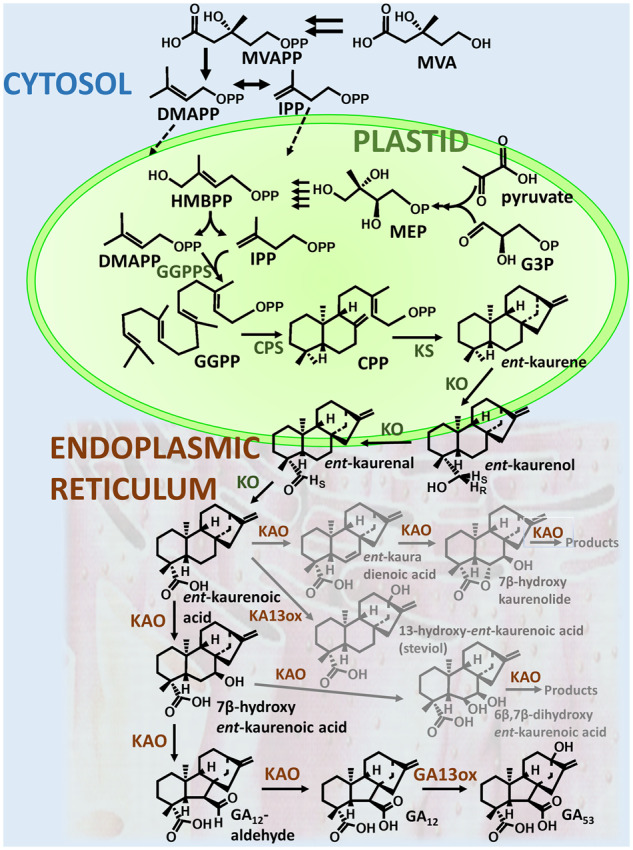
Early and middle sections of the GA-biosynthetic pathway indicating reactions in the cytosol, plastid and endoplasmic reticulum. Side reactions present in some developing seeds and the fungus *F. fujikuroi* are shown in gray. DMAPP, dimethylallyl diphosphate; G3P, glyceraldehyde 3-phosphate; HMBPP, 4-hydroxy-3-methylbut-2-enyl diphosphate; IPP, *iso*pentenyl diphosphate; MVAPP, mevalonate 5-diphosphate.

**Fig 3:**
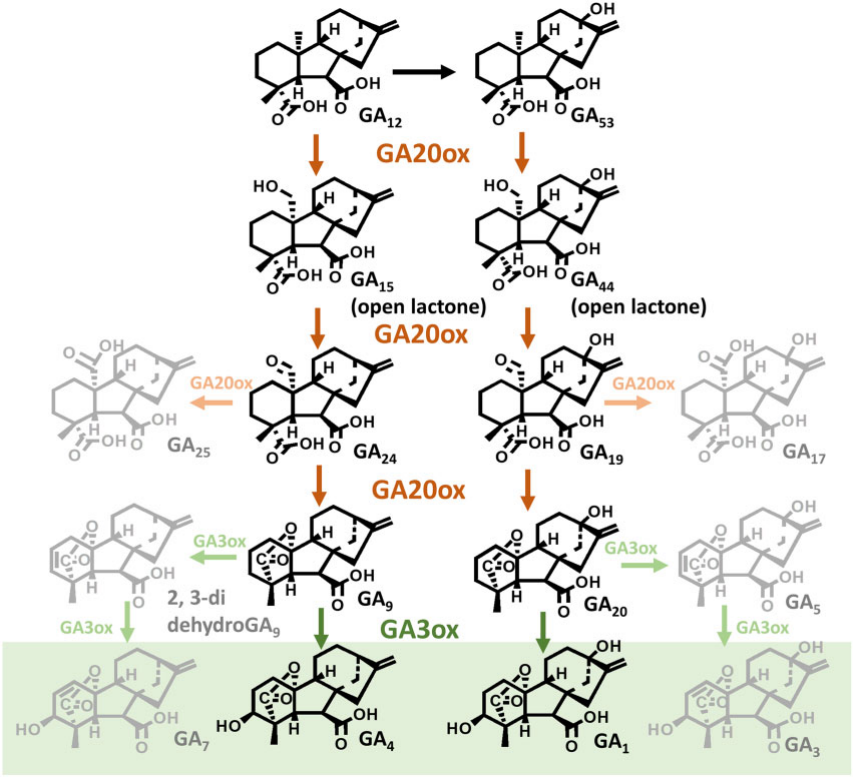
Final stage of GA biosynthesis to the biologically active end products GA_1_ and GA_4_ in plants catalyzed by the 2-oxoglutarate-dependent dioxygenases GA20ox and GA3ox. Reactions producing minor by-products of GA20ox activity (GA_25_ and GA_17_) and of GA3ox activity (GA_7_ and GA_3_), present in some species, are shown in gray. Biologically active GAs are highlighted in a green box.

## Formation of *ent*-Kaurene

As diterpenoids, GAs are formed from *trans*-geranylgeranyl diphosphate (GGPP), which is cyclized in two steps to the tetracyclic hydrocarbon precursor *ent*-kaurene via *ent*-copalyl diphosphate (Fig.�2). In plants, *ent*-kaurene is formed in plastids, predominantly via the methylerythritol 4-phosphate (MEP) pathway, although there is some contribution from the mevalonic acid (MVA) pathway, presumably dependent on the influx of isoprenoid intermediates of GGPP synthesis into the plastids from the cytosol (Kasahara et�al. 2002, Flugge and Gao 2005). Aach et�al. (1995)and Aach et�al. (1997) have shown that *ent*-kaurene formation from GGPP occurs in the stroma of proplastids or developing chloroplasts, but not in mature chloroplasts. The proplastid inner membrane is more amenable to the import of small molecules than is the chloroplast membrane (Brautigam and Weber 2009), which could also enable crossover between the two terpene pathways. In immature plastids, there is likely to be less competition for GGPP from the major pathways of chlorophyll and carotenoid formation than in chloroplasts. The question of how GGPP is allocated to the different pathways and the potential role of channeling has attracted some attention (Beck et�al. 2013, Ruiz-Sola et�al. 2016, Zhou et�al. 2017). Of the 10 functional GGPP synthase (GGPPS) genes in *Arabidopsis thaliana* (Arabidopsis), seven encode plastid-localized enzymes (Beck et�al. 2013), of which GGPPS11 is most strongly and constitutively expressed and is suggested by Ruiz-Sola et�al. (2016) to provide most of the substrate for the biosynthesis of photosynthesis-related terpenoids in this species. However, on the basis of gene co-expression networks and mutant analysis, Ruiz-Sola et al. concluded that GGPPS11 was unlikely to contribute to GA biosynthesis, for which one or more of the minor enzymes may be responsible. Interestingly, expression of four of these associated more closely with genes of the MVA pathway than with those of the MEP pathway, suggesting that they may obtain their substrates from the cytosol. Ruiz-Sola et�al. (2016) demonstrated that GGPPS11 interacted physically with enzymes involved in the biosynthesis of chlorophyll, carotenoids and plastoquinone and may form part of enzyme complexes, indicating the channeling of GGPP to these light harvesting components.

In contrast to Arabidopsis, rice is reported to contain one functional plastidic GGPPS, which must therefore be responsible for the biosynthesis of all diterpenoids in the plastid, including *ent*-kaurene (Zhou et�al. 2017). The rice GGPPS resides as a homodimer in the plastid stroma, but by forming a heterodimer with OsGRP (GGPPS recruiting protein), it is recruited to the thylakoid membrane, where it forms part of a protein complex involved in the biosynthesis of chlorophyll and other components of the light harvesting machinery. Distribution of GGPPS between the thylakoid and stroma (e.g. for the biosynthesis of GAs) would therefore depend on the abundance of OsGRP, which is related to the small subunit of type-II geranyldiphosphate synthases (GPS). In contrast to OsGRP, which would divert GGPPS from GA biosynthesis, a functional GPS is necessary for GA biosynthesis in tomato and Arabidopsis (van Schie et�al. 2007). The tomato GPS produced geranyl diphosphate and farnesyl diphosphate, but little GGPP from isopentenyl diphosphate and dimethylallyl diphosphate in vitro so must act in association with a GGPPS.

The two-step conversion of GGPP to *ent*-kaurene, proceeds by proton-initiated cyclization to the dicyclic *ent*-copalyl diphosphate (CPP) catalyzed by a type-II diterpene cyclase, *ent*-copalyl diphosphate synthase (CPS). Enzymes of this type contain a conserved DXDD motif, the middle aspartate donating a proton to initiate cyclization, while a water molecule coordinated to histidine and asparagine acts as the catalytic base to accept a proton and terminate the reaction (Koksal et�al. 2014, Lemke et�al. 2019). Using recombinant N-terminally truncated Arabidopsis CPS, Prisic and Peters (2007) demonstrated that enzyme activity was modified by Mg^2+^ and GGPP concentrations in a biphasic manner, with high concentrations of both inhibiting activity synergistically. As the concentration of Mg^2+^ and GGPP in plastids is promoted by light, Prisic and Peters (2007) suggested that this feedforward regulation of CPS activity reduced the flux into the GA pathway during deetiolation as part of the mechanism to decrease GA concentration. Mann et�al. (2010) noted that a conserved histidine residue in CPS enzymes involved in GA biosynthesis was associated with this inhibition by Mg^2+^, whereas type-II diterpene cyclases involved in secondary metabolism, which are less sensitive to Mg^2+^ inhibition, contain arginine at the equivalent position. The authors proposed that these basic residues act as a counter ion to the DXDD motif and differentially influence the binding of Mg^2+^ to this motif, which, while necessary for enzyme activity, is inhibitory at higher concentrations. The second step by which *ent*-copalyl diphosphate is converted to *ent*-kaurene is catalyzed by a type-I cyclase, *ent*-kaurene synthase (KS). In this reaction, cyclization is initiated by metal-dependent heterolytic cleavage of the C–O bond to form a pimeren-8-yl carbocation, which undergoes rearrangement and loss of H^+^ to form the tetracyclic *ent*-kaurene. (Zi et al. 2014). In common with other type-I terpene cyclases, KS contains DDXXD and RLX(N,D)DXX(S,T,G)XXX(E,D) motifs, which coordinate Mg^2+^ ions that associate with the diphosphate residue and participate in its ionization (Yamaguchi et�al. 1996, Zhou and Peters 2009, Liu et�al. 2014). Localization of CPS and KS in plastids was confirmed by Helliwell et�al. (2001) using protein-GFP fusions. Furthermore, CPS contains an N-terminal plastid-targeting sequence, which is cleaved on entry to the plastid, the resulting protein being more catalytically active in vitro than the uncleaved precursor (Sun and Kamiya 1994, Sun and Kamiya 1997). KS also contains a putative plastid-targeting sequence (Yamaguchi et�al. 1996, Yamaguchi et�al. 1998), although import into plastids has not been demonstrated.

In the moss *Physcomitrella patens*, which synthesizes *ent*-kaurenoic acid derivatives, but not GAs (Miyazaki et�al. 2018), CPS and KS activities are present as a single bifunctional protein containing both the DxDD and DDXXD motifs (Hayashi et�al. 2006). The lycophyte *Selaginella moellendorfii*, thought to be one of the earliest plants to have evolved the capability of synthesizing GAs, has monofunctional CPS and KS enzymes (Shimane et�al. 2014), which is a characteristic of vascular plants. While gymnosperms use bifunctional diterpene synthases to produce resin acids, *ent*-kaurene is formed by the two monofunctional enzymes (Keeling et�al. 2010). The more primitive KS activities in *P. patens* and *S. moellendorffii* were found to have relatively low substrate specificity, converting different stereoisomeric forms of CPP to a range of products, while angiosperm KS enzymes are specific for *ent*-CPP (Shimane et�al. 2014). In angiosperms, CPS and KS have undergone considerable gene expansion and functional diversification to produce diterpenoids involved in plant defence, as has been particularly well documented in cereals (Peters 2006, Xu et�al. 2007, Wu et�al. 2012, Zhou et�al. 2012, Fu et�al. 2016, Murphy et�al. 2018, Ding et�al. 2019).

In GA-producing bacteria and fungi, the genes for GA biosynthesis are clustered in operons, which in most cases include a *GGPPS* gene dedicated to the GA pathway (Malonek et�al. 2005, Nagel and Peters 2017). While the fungal operons contain a bifunctional CPS/KS, in bacteria these activities are separate, as in vascular plants (Morrone et�al. 2009). On the basis of conservation of the catalytic amino acid dyad in CPS between bacteria and plants, but not fungi, Lemke et�al. (2019) suggested a common origin of *ent*-kaurene synthesis for bacteria and plants, perhaps reflecting the endosymbiotic origin of chloroplasts. The bacterial and plant enzymes share some sequence and structural homology, but the bacterial enzymes are smaller than those in plants. As discussed by Morrone et�al. (2009), fusion between CPS and KS may have occurred in early plant evolution, resulting in the bifunctional CPS/KS in bryophytes, such as *P. patens*, with gene duplication and respective loss of CPS or KS activities to produce separate enzyme activities occurring in lycophytes. This is supported by the considerable sequence homology between CPS and KS in vascular plants (Yamaguchi et�al. 1996). As noted above, bifunctional diterpene synthases have been retained in conifers, but not for GA biosynthesis.

## Formation of C_20_-Gibberellins

The conversion of *ent*-kaurene to GA_12_, the first C_20_-GA on the biosynthetic pathway, is catalyzed by two cytochrome P450 monooxygenases, *ent*-kaurene oxidase (KO) and *ent*-kaurenoic acid oxidase (KAO) (Helliwell et�al. 1999, Helliwell et�al. 2001) (see Fig.�2). Helliwell et�al. (2001) showed that the Arabidopsis KO associates with the outer chloroplast membrane and possibly also the endoplasmic reticulum (ER), while the two Arabidopsis KAOs were located at the ER. This suggests that *ent*-kaurene is oxidized as it exits the plastid onto the ER, potentially through a membrane connection that allows the trafficking of nonpolar metabolites between the plastid and the ER (Mehrshahi et�al 2013, Block and Jouhet 2015). KO, which in plants belongs to the subfamily CYP701A, catalyzes the three-step oxidation of *ent*-kaurene to *ent*-kaurenoic acid by repeated hydroxylation of C-19, with the intermediate diol apparently undergoing dehydration to form the aldehyde (*ent*-kaurenal) before further hydroxylation to *ent*-kaurenoic acid (Morrone et�al. 2009, Nagel and Peters 2017). On the basis of kinetic analysis, it was concluded that the two intermediates in the reaction remain at the enzyme active site and that the first hydroxylation to *ent*-kaurenol is the rate-limiting step (Morrone et�al. 2009). In common with CPS and KS, the KO-like genes have proliferated in cereals with functional diversification to produce diterpenoids involved in plant defence. Rice has five KO-like genes arranged in tandem on chromosome 6, of which *OsKO1*, *OsKO2* and *OsKO5* (encoding CYP701A7, CYP701A6 and CYP701A9, respectively) are involved in GA biosynthesis (Itoh et�al. 2004, Sakamoto et�al. 2004, Chen et�al. 2019, Zhang et�al. 2020), while OsKO4 (CYP701A8) hydroxylates *ent*-kaurene and related diterpenes at the 3α position, which is in close proximity to C-19 (Wang et�al. 2012). By coupling the N-terminal regions to GFP, Zhang et�al. (2020) showed that OsKO2, similar to AtKO, is located in the plasmid outer membrane, whereas OsKO1 is present in the plastid and endomembranes. While the wheat KO family has not been functionally characterized, it is of interest to note that treatment of wheat seedlings with the KO-inhibitor paclobutrazol resulted in the accumulation inter alia of 3α-hydroxy-*ent*-kaurene (Croker et�al. 1995), which is probably a product of a KO paralog. The fungal and bacterial KOs belong to the CYP503 (Tudzynski et�al. 2001) and CYP117 (Nett et�al. 2017) families, respectively, and are not closely related to the plant enzymes or to each other.

The reaction sequence catalyzed by KAO has been studied mainly in cell-free systems from developing seeds, particularly from endosperm of Cucurbitaceae, and from cultures and cell-free systems from the fungus *F. fujikuroi* (reviewed in Hedden and Sponsel 2015). In plants, the product of KAO (CYP88A subfamily) is GA_12_, which is formed from *ent*-kaurenoic acid in three steps via 7β-hydroxy-*ent*-kaurenoic acid and GA_12_-aldehyde. The first step involves stereospecific hydroxylation on C-7β, while the second reaction, in which ring B contracts from 6 to 5 carbon atoms through migration of the C-7–C-8 bond from C-7 to C-6 with the resulting extrusion of C-7 as the aldehyde, is initiated by stereospecific loss of the 6β-H (Graebe et�al. 1975, Castellaro et�al. 1990). In the final step, GA_12_-aldehyde is oxidized to GA_12_. The Cucurbitaceae *Cucurbita maxima* (pumpkin) and *Cucumis sativa* (cucumber) contain GA 7-oxidases (GA7ox), which are soluble 2-oxoglutarate-dependent dioxygenases (2-ODDs) that convert GA_12_-aldehyde to GA_12_, although they also have other activities. They are present particularly in developing seeds (Lange et�al. 1994, Frisse et�al. 2003, Lange et�al. 2013) but are also expressed in vegetative tissues, including the roots (Lange et�al. 2005, Sun et�al. 2018). The species distribution of GA7ox appears to be restricted, with apart from the Cucurbitaceae, two reports of their presence in potato (Fixen et�al. 2012, Katsarou et�al. 2016).

The fungal KAO (belonging to the CYP68A subfamily) produces GA_14_ rather than GA_12_ by catalyzing an additional 3β-hydroxylation (Rojas et�al. 2001). The substrate for this reaction is thought to be GA_12_-aldehyde, to produce GA_14_-aldehyde, and not GA_12_ as, in contrast to GA_14_-aldehyde and GA_14_, GA_12_ is not converted to 3β-hydroxyGAs by fungal cultures (Bearder et�al. 1975). The seed and fungal KAOs possess remarkable multifunctionality, producing numerous by-products, of which the seco-ring B compounds fujenal and fujenoic acid are major metabolites (Rojas et�al. 2001). Fujenal results from oxidative ring cleavage of 6β,7β-dihydroxy-*ent*-kaurenoic acid, which is formed from 7β-hydroxy-*ent*-kaurenoic acid by stereospecific hydroxylation at C-6β (Castellaro et�al. 1990). Ring contraction and hydroxylation are, thus, competing outcomes following the initial removal of the 6β-H to form a radical or carbocation (Graebe et�al. 1975, Nett et�al. 2016). A second group of by-products are the kaurenolides, which contain a C-19,6α lactone and a 7β-hydroxy group. They are formed from *ent*-kaurenoic acid via *ent*-kaura-6,16-dienoic acid, which is proposed to be converted to 7β-hydroxykaurenolide by nonenzymatic reaction of the C-4α carboxylate (C-19) with an intermediate 6β,7β-epoxide (Hedden and Graebe 1981, Beale et�al. 1982). Dehydrogenation at C-6,7 in the formation of *ent*-kaura-6,16-dienoic acid occurs with stereospecific removal of 7β-H, but non-stereospecific loss of H from C-6 (Castellaro et�al. 1990). As these *ent*-kaurenoid by-products are not converted to GAs, their formation detracts seriously from GA formation. However, it is notable that they are formed in plant organs and the fungus, which produce large quantities of GAs and related *ent*-kaurenoids with uncertain function in the producing organs/organism. Only 7β-hydroxy-*ent*-kaurenoic acid and GA_12_ were formed when KAO from Arabidopsis and barley was incubated with *ent*-kaurenoic acid after expression in yeast (Helliwell et�al. 2001). It is possible that KAOs in plant tissues that produce GAs in hormonal quantities exert much tighter catalytic control over the reaction, or that the reaction outcome is sensitive to substrate concentration.

In the bacterial GA operon, a CYP114 family P450 is responsible for the KAO activity (Nagel and Peters 2017, Nett et�al. 2017). It converts *ent*-kaurenoic acid only to GA_12_-aldehyde, which is oxidized to GA_12_ by a short-chain alcohol dehydrogenase, encoded from the operon. Full CYP114 activity is dependent on electrons from a dedicated ferredoxin within the operon, although the first reaction (7β-hydroxylation) can proceed when this is not present (Nett et�al. 2016). There is no evidence that CYP114 produces products other than GA_12_-aldehyde.

## Gibberellin Biosynthesis from GA_12_

The formation of the bioactive end products of the pathway from GA_12_ involving mainly 2-ODD enzymes in plants is illustrated in Fig.�3. The pathway branches from GA_12_, 13-hydroxylation to GA_53_ initiating the formation of 13-hydroxylated GAs, such as GA_1_, while a parallel non-13-hydroxylation pathway from GA_12_ results in GA_4_ formation.

### GA 13-hydroxylation and related activities

13-Hydroxylation of GA_12_ in rice was shown by Magome et�al. (2013) to be catalyzed by two cytochrome P450s, CYP714B1 and CYP714B2. Although vegetative tissues of rice contain predominantly 13-hydroxylated GAs (Kobayashi et�al. 1988), the overexpression of either *CYP714B* gene in rice, which resulted in an increase in the concentration of 13-hydroxy GAs, including GA_1_, also caused semidwarfism (Magome et�al. 2013). This finding prompted Magome et al. to suggest that 13-hydroxylation was a mild inactivating reaction that adjusted the balance of bioactive GAs in favor of GA_1_ relative to the more active GA_4_. Higher biological activity of GA_4_ is in accord with the properties of the rice GA receptor, GID1, which has a greater affinity for GA_4_ than for the 13-hydroxy GAs GA_1_ and GA_3_ (Ueguchi-Tanaka et�al. 2005), although GA_1_ and GA_4_ have similar activities in rice bioassays, while GA_3_ is considerably more active (Crozier et�al. 1970, Nishijima and Katsura 1989). This anomaly may be related to differences in the efficiency of transport, or inactivation between the GAs when applied in bioassays.

Arabidopsis contains two members of the CYP714A subfamily, which are expressed in developing seeds (Zhang et�al. 2011, Nomura et�al. 2013). CYP714A1 converts GA_12_ to 16α-carboxy-17-norGA_12_ and caused severe dwarfism when overexpressed in Arabidopsis. CYP714A2, which caused mild dwarfism when overexpressed, converts GA_12_ to 12α-hydroxyGA_12_ (GA_111_) and, to a small extent, GA_53_ (by 13-hydroxylation) and also 13-hydroxylates *ent*-kaurenoic acid to form steviol (Nomura et�al. 2013). Another CYP714 family member, CYP714D1, known also as ELONGATED UPPERMOST INTERNODE (EUI), present in rice, epoxidizes the 16,17-double bond of 13-deoxyGAs, including GA_12_, to form inactive products (see below) (Zhu et�al. 2006). Thus, CYP714 family members have a generally GA-inactivating function by oxidizing GAs and/or *ent*-kaurenoids on the C and D rings (see below). It was reported recently that some members of the CYP72A subfamily have a similar function (He et�al. 2019). Arabidopsis contains eight tandem CYP72A genes, one of which, CYP72A9, was shown by heterologous expression in yeast to 13-hydroxylate GA_12_ as well as GA_9_ and GA_4_. It also acted on *ent*-kaurenoic acid, but in this case 13-hydroxylation was a minor activity, the major product being the 16α,17-dihydroxy derivative, presumably formed via epoxidation of the 16,17-double bond. Most Arabidopsis organs contain much higher amounts of GA_4_ than of GA_1_, the exception being developing seeds, in which the CYP72A genes are most highly expressed. He et�al. (2019) suggested that CYP72A9 and some paralogs may be the major source of 13-hydroxy GAs in the seed. They determined the function of CYP72A family members in Arabidopsis and other species and showed that some have related activities, although usually with a more restricted substrate range. Indeed, many of the enzymes for which activity could be shown acted only on *ent*-kaurenoic acid. Overexpression of *CYP72A9*, but not its paralogs, in Arabidopsis resulted in strong dwarfism, while seeds of *cyp72a9* mutants germinated more rapidly than those of the wild type without stratification, suggesting that CYP72A9, in common with other GA-inactivating enzymes in seeds, may have a role in promoting dormancy (He et�al. 2019). Low levels of 13-hydroxylase activity have also been noted for certain 2-ODDs for which the primary function is GA 3β-hydroxylase activity, such as the wheat enzyme TaGA3ox2 (Appleford et�al. 2006) and MmGA3ox2 from *Marah macrocarpus* (Ward et�al. 2010). As C-3 and C-13 are spatially widely separated on the GA molecule, 13-hydroxylation requires that the orientation of the substrate in the active site is rotated horizontally from that required for 3β-hydroxylation. Interestingly, a 2-ODD from *Tripterygium wilfordii* functions as a 13-hydroxylase, converting GA_9_ to GA_20_, but did not act on GA_4_, while other substrates were not tested (Zhang et�al. 2019). Phylogenetic analysis showed that the *T. wilfordii* enzyme is related most closely to the GA 3-oxidases (GA3ox).

A novel 2-ODD activity acting on GA_12_ in Arabidopsis was reported recently by two groups (Xiong et�al. 2018, Liu et�al. 2019). The gene, named *GIM2* (Xiong et�al. 2018) or *GAS2* (Liu et�al. 2019), both corresponding to At2g36690, encodes a 2-ODD and promotes seed germination when overexpressed. It was shown by both groups that the enzyme expressed in *Escherichia coli* acts on GA_12_, but in one case it produced an unidentified hydroxyGA_12_ (Xiong et�al. 2018), while in the other it hydrated the 16,17-double bond (Liu et�al. 2019), which is an unusual 2-ODD activity. Overexpression of *GIM2* produced on overall increase in the GA content including that of GA_4_ in germinating seed, with reduced GA levels in a *gim2* mutant, while *GAS2* overexpression reduced the GA_4_ content in seeds while increasing the concentration of hydrated GA_12_. As both groups were working with the same gene, the discrepancy in their results is difficult to explain. There must still be some uncertainty about the function of this enzyme and whether GA_12_ is its only substrate.

### GA 20-oxidases

The conversion of GA_12_ and GA_53_ to GA_9_ and GA_20_, respectively, is catalyzed in plants by a family of 2-ODDs, known as GA 20-oxidases (GA20ox), that cleave C-20 with the formation of the 19,10-γ-lactone characteristic of C_19_-GAs. In the reaction sequence, the C-20 methyl is oxidized to the alcohol and then to the aldehyde, from which C-20 is lost. The alcohol and aldehyde intermediates accumulate and are efficiently converted further, which is consistent with the mechanism of 2-ODD enzymes, in which the reaction products are released from the enzyme active site before the substrates are rebound for the next round of oxidation (Myllyla et�al. 1977). The alcohol intermediates, i.e. GA_15_ and GA_44_ for the 13-H and 13-OH forms, respectively, are isolated as the 19,20-δ-lactones from plant tissues, probably due to spontaneous lactonization under the low pH conditions necessary to extract them. Most plant GA20ox enzymes preferentially accept the free C-20 alcohol as substrate, suggesting that in vivo the intermediates are present at least to some extent as the free alcohols as would be anticipated at the cytosolic pH of >7. An uncharacterized activity that oxidizes the δ-lactone to the aldehyde has been described, such as that found in spinach (Gilmour et�al. 1986). While oxidation of the C-20 alcohol by GA20ox involves stereospecific loss of the 20-*pro*R H, the spinach enzyme removed the 20-*pro*S H from the lactone, in which the 20-*pro*R H is fixed in a sterically hindered and poorly accessible position (Ward et�al. 1997). The mechanism for the loss of C-20 from the aldehyde is not well understood. The 20-oic acid is normally a minor by-product of the reaction (Lange et�al. 2013), except in the case of a GA20ox from *C. maxima* seeds, for which it is the main product (Lange et�al. 1994). However, this carboxylic acid is not an intermediate in C_19_-GA biosynthesis (Kamiya and Graebe 1983). Nevertheless, C-20 is lost as CO_2_ rather than as formic acid, which would be the case if it was lost directly from the aldehyde (Kamiya et�al. 1986). Ward et�al. (2002) provided evidence consistent with the reaction proceeding via a C-10 radical, which it was suggested reacts with the 19-oic acid group to form the 19,10-lactone. The mechanism by which this radical is formed is unclear. In contrast to the alcohol and aldehyde intermediates, which accumulate often to relatively high concentrations, no intermediate between the aldehyde and C_19_-GA final product has been identified, prompting Ward et al. to suggest that the intermediate may be enzyme-bound, although there is as yet no supporting evidence.

In fungi and bacteria, C-20 oxidation is catalyzed by the cytochrome P450s CYP68B and CYP112, respectively (Nagel and Peters 2018), which perform the same sequence of reactions as the plant GA20ox. It has not been possible to study the fungal enzyme in vitro, but work with cultures of the *F. fujikuoi* mutant B1-41a indicates that the intermediates are not released from the enzyme since they do not accumulate and are converted inefficiently to C_19_-GAs compared with earlier precursors when supplied to cultures (Bearder et�al. 1975). Through the use of ^18^O-labeled substrates, it was also established that both O atoms in the 19,10-lactone originate from the 19-oic acid (Bearder et�al. 1976). In contrast to the fungal enzyme, CYP112 from the bacterium *Erwinia tracheiphila*, prepared by expression in *E. coli*, converted GA_12,_ GA_15_ (open and closed lactone) and GA_24_ to GA_9_ and accumulated the intermediates when incubated under NADPH-limiting conditions (Nagel and Peters 2018). On the basis of incubations in ^18^O_2_, Nagel and Peters (2018) confirmed that C-20 is lost as CO_2_ and proposed a mechanism in which the aldehyde, GA_24_, present as the C-20 geminal diol or as the lactol, is oxidized to the C-19–20 anhydride with C-20 present as the geminal diol. Further oxidation of this intermediate would release C-20 as CO_2_ forming the 19,10-lactone by rearrangement via the C-10 radical. While it is possible that hydrolysis of the anhydride to the dicarboxylic acid could account for the small amounts of 20-oic acid formed as a by-product of C_19_-GA formation in plants, it is also noteworthy that C-19,20 dioic acids readily form the anhydride in solution but are not substrates for GA20ox in plants, fungi or bacteria (Kamiya and Graebe 1983, Tudzynski et�al. 2002, Nagel and Peters 2018).

Seed plants contain a family of *GA20ox* genes, with members differing in their developmental, environmental and tissue expression patterns. For example, Arabidopsis contains five *GA20ox* genes encoding functionally similar enzymes, except for AtGA20ox5, which produces the aldehyde without further conversion to the C_19_-GA (Plackett et�al. 2012). *AtGA20ox1* and *AtGA20ox2* act partially redundantly in plant development, with *AtGA20ox3* having a minor role, while the physiological function of the other two genes is unclear (Rieu et�al. 2008, Plackett et�al. 2012). Poaceae (grasses), including the cereals, typically contain four *GA20ox* genes, with the expression of one of them, *GA20ox3*, although relatively very high, restricted to the endosperm of developing grain, which produces large amounts of GAs of uncertain function (Pearce et�al. 2015). It should be noted that gene annotation numbers, which usually relate to their order of discovery, do not denote orthology, except within plant families, as GA-oxidase gene multiplication and divergence seems to have occurred relatively late in evolution (Han and Zhu 2011, Huang et�al. 2015). In many species, GA20ox activity limits the GA production (Fleet et�al. 2003) and expression of *GA20ox* paralogs with major developmental roles is tightly regulated by developmental and environmental signals and by GA signaling to maintain GA homeostasis (see below).

### GA 3-oxidases

In the final step in the biosynthesis of bioactive GAs, the C_19_-GAs GA_9_ and GA_20_ are 3β-hydroxylated to GA_4_ and GA_1_, respectively, by GA 3-oxidase (GA3ox) enzymes. GA3ox genes that are expressed in vegetative tissues of eudicots generally function only as 3β-hydroxylases with high regiospecificity, while those in monocots are less regiospecific, so that, e.g. GA_3_ is produced from GA_20_ as a minor by-product of GA_1_ production (see Fig.�3; Itoh et�al. 2001). This side reaction occurs by oxidation at both the 2β and 3β positions to form the 2,3-unsaturated intermediate GA_5_, which is converted to GA_3_ by oxidation on C-1β by the same enzyme, followed by the migration of the double bond to the 1,2 position and hydroxylation on C-3β, (Albone et�al. 1990, Fujioka et�al. 1990). Thus, monocots contain low levels of GA_3_, usually <10% of the GA_1_ content, while it is usually undetectable in vegetative tissues from eudicots. GA3ox-like enzymes present in developing seeds of both eudicots and monocots may have quite diverse activities. For example, a GA3ox from *C. maxima* seed acts also on C_20_-GAs (Frisse et�al. 2003) and two GA3ox-like enzymes act in sequence to produce GA_7_ in *M. macrocarpa* seeds via 2,3-didehydroGA_9_ (see Fig.�3) (Ward et�al. 2010) and GA_54_ (1β-hydroxyGA_4_) via GA_61_ in wheat endosperm (Pearce et�al. 2015). The wheat enzymes are closely related paralogs produced by a recent gene duplication with functional diversification such that, while one (TaGA3ox3) retains 3β-hydroxylase activity, the other acts as a 1β-hydroxylase and is annotated as TaGA1ox1 (Pearce et�al. 2015). Barley contains an ortholog of these enzymes that hydroxylates at both the C-3β and C-18 positions to convert GA_9_ to GA_131_ (18-hydroxyGA_4_)(Pearce et�al. 2015). GA_54_ and GA_131_ are the major GAs present in developing seeds of wheat and barley, respectively (MacMillan 2001), although their function in seeds is unknown. GA biosynthesis is often very strong in developing seeds, which can produce a wide array of structures, reflecting the functional diversity of the enzymes involved, although in many cases the nature of these enzymes is still unknown.

*GA3ox* genes are present as small families, with Arabidopsis containing four members and rice and barley only two. Two of the Arabidopsis enzymes, AtGA3ox1 and AtGA3ox2, and only one in cereals, GA3ox2, have major roles in the development of vegetative organs. A second rice enzyme OsGA3ox1, which contributes particularly toward reproductive development, does not have close orthologs in barley and wheat, in which, apart from GA3ox2, the other GA3ox-like genes are mainly expressed in developing seeds.

In *F. fujikuroi* and other related GA-producing *Fusarium* species, 3β-hydroxylation occurs early in the pathway catalyzed by the highly multifunctional P450-1 (CYP68A) (see above). A close ortholog has the same function in the Cassava pathogen *Sphaceloma manihoticola*, which produces GA_4_ (B�mke et�al. 2008), while the GA_1_-producing *Phaeosphaeria* spp. utilizes an unrelated CYP to convert GA_9_ to GA_4_ in a pathway resembling that in plants (Kawaide 2006). In *F. fujikuroi*, the last two steps in GA_3_ biosynthesis are the desaturation of GA_4_ to GA_7_, catalyzed by a 2-ODD, DES (Bhattacharya et�al. 2012), and 13-hydroxylation, catalyzed by P450-3 (Tudzynski et�al. 2003). The genes encoding DES and P450-3 lie on opposite ends of the GA operon in *F. fujikuroi* and are missing in the *S. manihoticola* operon. In the GA-biosynthetic operons of GA_4_-producing plant pathogenic bacteria, such as *Xanthomonas oryzae*, *CYP115* encodes a 3β-hydroxylase that converts GA_9_ to GA_4_ (Nagel et�al. 2017). However, the operons of most GA-producing symbiotic rhizobia lack CYP115 so that these bacteria produce GA_9_, which must be converted to GA_4_ by the host plant (Nagel and Peters 2017), although some rhizobia contain CYP115 and are able to produce GA_4_ (Nett et�al. 2017). Production of bioactive GAs by fungal and bacterial plant pathogens is proposed to facilitate infection by suppressing jasmonic acid signaling that promotes immunity, whereas in the symbiotic N_2_-fixing rhizobia, in which GA has a role in nodule formation, the ability of the plant to regulate GA production must be advantageous (Nagel and Peters 2017).

## Inactivation

### GA 2-oxidases

Inactivation, i.e. introducing structural modifications that decrease affinity for the receptor, is an essential activity to regulate the concentration of biologically active GAs in plant tissues. A number of inactivating reactions have been described (illustrated in Fig.�4), the most universal being 2β-hydroxylation, which can occur on the bioactive end products of the pathway or on the C_19_- or C_20_-GA precursors, so preventing formation of the active species. Gibberellin 2-oxidases (GA2ox) form two major families of 2-ODDs, consisting of enzymes that act primarily on C_19_-GA substrates and those that act mainly on C_20_-GAs. While these families are phylogenetically not closely related, there is functional overlap, with some enzymes belonging to the C_19_-GA2ox family acting on C_20_-GAs, usually as a minor activity, and vice versa (Lange et�al. 2013, Pearce et�al. 2015). The C_19_-GA2ox family is the largest of the GA 2-ODD families and, on the basis of sequence, falls into two sub-families, this division preceding the divergence of the monocots and eudicots (Kawai et�al. 2014, Huang et�al. 2015). While *GA2ox* genes are present in seed plants (Niu et�al. 2014, Huang et�al. 2015), 2β-hydroxyGAs are apparently not produced in lycophytes and ferns, suggesting the absence of functional *GA2ox* genes, which therefore evolved later than the other GA 2-ODDs. 2β-HydroxyGAs are also not produced by fungi and bacteria (MacMillan 2001), in which GAs do not have a developmental role so that precise regulation of their concentration may not be critical. The exceptions are symbiotic GA-producing bacteria for which GA production may need to be more tightly regulated, but as noted above, they produce the precursor, GA_9_, allowing the plant host to regulate the synthesis of bioactive GA. Some C_19_-GA2ox enzymes have been shown to oxidize the 2β-hydroxyGA product further to the 2-ketone (Thomas et�al. 1999). These products are isolated as the so-called ‘GA catabolites’, in which the 19,10-lactone has opened with formation of a double bond between C-10 and C-1 (Gaskin et�al. 1981). It is likely that the catabolites are artifacts of isolation and that the product in planta is the unrearranged ketone.

**Fig 4:**
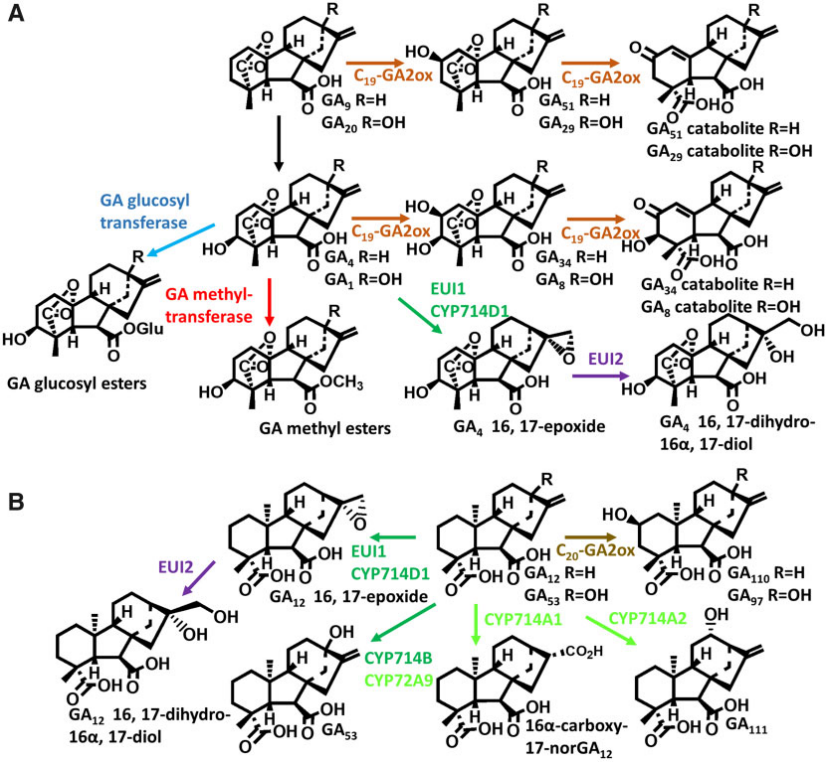
Reactions involved in GA inactivation, acting on C_19_-GAs (A) or C_20_-GAs (B). Different enzyme types are indicated by color: brown, 2-oxoglutarate-dependent dioxygenases; green, cytochrome P450s (enzymes present in rice in dark green and in Arabidopsis light green); red, methyltransferases; blue, glucosyltransferases; and purple, epoxide hydrolase. GA_12_ 13-hydroxylation, which initiates the biosynthesis of GA_1_, results in slight reduction in bioactivity and is consequently included in (B). The involvement of EUI2 in dihydrodiol formation is assumed, but has not been demonstrated.

The recent determination of the X-ray crystal structure of the rice C_19_-GA2ox OsGA2ox3 revealed that it formed a tetramer in the presence of its substrate GA_4_, with the monomers linked via two GA_4_ molecules and two disulfide bridges (Takehara et�al. 2020). The tetramer was shown to be more active than the monomer, exhibiting a lower Km for GA_4_ by enabling an energetically more favorable pathway to the active site. Thus, increasing GA_4_ concentration promotes multimer formation and enhances enzyme activity, providing an allosteric feedforward mechanism to maintain GA homeostasis. GA_4_ also promoted dimerization of the C_20_-GA2ox OsGA2ox6, for which it is not the preferred substrate. Remarkably, Takehara et�al. 2020 found a similar mechanism, involving dimerization of the auxin catabolic enzyme indoleacetic acid oxidase, also a 2-ODD, in the presence of its substrate.

GA2ox enzymes have an essential function in regulating GA concentration during normal plant development, and also in response to changes in environmental conditions. The expansion of the GA2ox families has enabled some specificity in gene expression at the tissue/organ level (Li et�al. 2019) and in response to stress (Colebrook et�al. 2014), although there is considerable redundancy (Ross and Reid 2010). In their assessment of GA-metabolic gene specialization in rice through CRISPR/Cas9 gene knock-out, Chen et�al. (2019) found that two of the three C_20_-GA2ox genes have specific roles in fertility and grain development, whereas loss of individual genes had no effect on stem extension, potentially due to redundancy. The wheat reduced height alleles *Rht18* and *Rht14* cause increased expression of the C_20_-GA2ox gene *TaGA2oxA9* in stems, while some loss-of-function *taga2ox9* mutants generated from *Rht18* exhibited overgrowth phenotypes compared with the tall parent of *Rht18*, which suggests a potential role for this gene in the control of stem height (Ford et�al. 2018). Increased internode length in tomato was also reported for a mutant of the tomato C_20_-GA2ox gene *SlGA2ox7* (Schrager-Lavelle et�al. 2019). The reduction in the bioactive GA content in leaves as they mature was shown in pea to be due to high rates of 2-oxidation rather than reduced biosynthesis (Ross et�al. 2003). In developing seeds, GA2ox activity may increase to high levels as the seed approaches maturity (Albone et�al. 1984), ensuring that bioactive GAs do not accumulate in mature seed and thereby induce precocious germination and/or abnormal seedling growth. This is illustrated by the *slender* (*sln*) mutant of pea, which has an overgrowth phenotype during early seedling development due to a mutation in the *PsGA2ox1* gene that allows GA_20_ to accumulate in the mature seed (Lester et�al. 1999). Conversion of GA_20_ to GA_1_ following seed imbibition promotes the excessive seedling growth. It has been reported for several species that *GA2ox* expressed at the base of the shoot apical meristem limits the influx of bioactive GA to the meristem to control meristem function (Sakamoto et�al. 2001, Jasinski et�al. 2005, King et�al. 2008, Bolduc and Hake 2009). Induction of *GA2ox* expression by stress is a common mechanism for growth control and enhanced stress tolerance with different *GA2ox* genes being targeted according to the stress (reviewed in Colebrook et�al. 2014). In a recent example, touch-induced growth reduction in Arabidopsis was associated with increased expression of *AtGA2ox7*, a C_20_-GA2ox gene (Lange and Lange 2015).

### Other inactivation mechanisms

As described above, cytochrome P450s belonging to the CYP714 family have a generally inactivating activity by oxidizing GAs on the C and D rings (see Fig.�4B). Of particular significance, EUI1 (CYP714D1) has an important developmental function in rice by restricting culm height, acting particularly on the upper internodes (Zhu et�al. 2006). Introduction of *eui1* mutant alleles into male sterile rice to allow adequate panicle exsertion was an important development for hybrid rice production (Liang et�al. 2008). EUI1 acts on 13-H GAs to form 16α,17-epoxides, although 16α,17 diols rather than the epoxides are present in planta, and these are likely to be formed from the epoxides by nonenzymatic or enzymatic hydrolysis. Rice contains a second *EUI* gene, *EUI2*, encoding an α,β-hydrolase (Zhu 2003), which may have this function. GA 16,17-dihydrodiols are widely distributed in plants suggesting that double bond epoxidation and hydrolysis may be a common activity. *EUI1* is highly expressed in the nodes and intercalary meristem of the upper internodes, as well as in the flowering spikelets of the young panicle (Zhu et�al. 2006). Unlike vegetative tissues of rice, reproductive tissues contain predominantly 13-H GAs, with mature anthers containing very high concentrations of GA_4_ (Hirano et�al. 2008). As EUI is active only against 13-H GAs, it is tempting to suggest that it regulates the levels of GA (GA_4_ or precursors) reaching the upper internodes by movement from the panicle, a process that serves to coordinate panicle exsertion with anthesis.

GA inactivation by methylation, catalyzed by members of the SABATH family of methyl transferases, has been reported for Arabidopsis, in which the enzymes are present in developing seed (Varbanova et�al. 2007, Xing et�al. 2007). Although they have been described only for Arabidopsis, GA methyl transferases are more widely distributed in seed plants and are also present in some fern species, in which methyl GAs are present as antheridiogens (Yumane et�al. 1988, Tanaka et�al. 2014). Sugar conjugation, particularly reversible esterification with glucose (Schneider et�al. 1992), is also a potential inactivation mechanism. There has been a hiatus in research on GA conjugates since the 1990s, but their relevance to GA metabolism and transport merits renewed investigation.

## Sites of GA Biosynthesis and GA Mobility

The sites of GA synthesis and their relationship to the sites of action are of major relevance to any consideration of function. There is renewed interest in GA distribution stemming from the identification of GA transporters and the development of in vivo methods to determine GA distribution and movement at the cellular level (Rizza et�al. 2017, Wexler et�al. 2019). The topic has been reviewed recently (Lacombe and Achard 2016, Binenbaum et�al. 2018, Rizza and Jones 2019) and will be discussed only briefly here. The sites of GA synthesis are usually inferred from the expression of biosynthesis genes on the basis of reporter activity, in situ hybridization or, in the case of Arabidopsis roots, transcript analysis in combination with cell isolation and sorting (Birnbaum et�al. 2003, Dugardeyn et�al. 2008). However, this does not allow for differences in translational efficiency or enzyme stability. Treatment of spinach with GA biosynthesis inhibitors resulted in elevated levels of SoGA20ox1 protein, measured by Western blotting, in the petioles and shoot tip, but no change in the transcript level (Lee and Zeevaart 2007), emphasizing the need to consider posttranscriptional regulation. The location of GA biosynthesis has been investigated more directly from the application of radioactively labeled GAs, e.g. in pea (Smith 1992, O’Neill and Ross 2002), but such studies provide limited spatial resolution. Normal development under non-stressful conditions depends on appropriate coordination of GA biosynthesis and inactivation. Reinecke et�al. (2013) were able to complement the dwarf phenotype of the pea *ga3ox1* (*le*) mutant more effectively by introgressing the native *PsGA3ox1* gene than by constitutive expression of its cDNA from the 35S promoter. Ectopic expression of *PsGA3ox1*, which is rate limiting for GA biosynthesis in pea, resulted in strong upregulation of the GA-catabolic gene *PsGA2ox1*, which the authors suggested would be normally segregated from cells responsible for GA biosynthesis.

In vegetative organs, GAs are synthesized mainly in growing regions, such as elongating stems and leaves, and root tips. Very high rates of synthesis occur in certain tissues, including anthers (Hirano et�al. 2008) and the cereal scutellar epithelium (Kaneko et�al. 2003), which act as sources for other tissues (see below), and in developing seeds, in which the function of GA is unclear. While there is evidence based on transcript localization for GA biosynthesis occurring at or close to the site of action, e.g. in cereal stems (Kaneko et�al. 2003, Pearce et�al. 2011) or Arabidopsis roots (Dugardeyn et�al. 2008), there are also examples of mobility between organs, where tissues act as a source of GA for neighbouring GA-nonautonomous organs. Examples are the cereal embryo scutellum as a source of GA for the aleurone (Appleford and Lenton 1997), the suspensor as a GA source for the embryo in several species (reviewed in Jacob and Brian 2020) and GA or precursors from the anther tapetum being required for filament elongation and petal growth (Weiss and Halevy 1989, Silverstone et�al. 1997, Hu et�al. 2008). In female cucumber flowers, GA_9_ produced in ovaries moves to the petals and sepals where it is converted to GA_4_, which promotes the expansion of these organs (Lange and Lange 2016). In these cases, the hormone acts to coordinate the growth and development of neighboring, physiologically related organs. As suggested above, GA from the anthers in cereals may also stimulate peduncle elongation to ensure adequate emergence of the spike. There are also examples of long distance GA transport, such as from leaves to induce the transition to flowering at the shoot apex in Arabidopsis (Eriksson et�al. 2006) and *Lolium* (King et�al. 2001). In some cases, long distance movement of precursors rather than the active hormone has been noted (Proebsting et�al. 1992, Regnault et�al. 2015). It is unclear what specifies the structure of the mobile molecules, but it may be determined by the properties of transmembrane transporters. So far only influx transporters have been identified and these lack specificity, transporting other hormones as well as unrelated molecules (reviewed in Binenbaum et�al. 2018). According to the ion-trap hypothesis, the high pH environment of the cytosol would deter efflux of GAs by passive diffusion through the cell membrane, while influx from the more acidic apoplast would be more favored (Kramer 2006). However, passive diffusion across biological membranes, which are rich in proteins and other molecules that can interact with mobile signals, may be limited (Kell 2015), such that both influx and efflux transporters are necessary for effective mobility. The identification and properties of GA transporters is likely to remain an active field of research.

## Regulation of GA Metabolism

The concentration of biologically active GAs in GA-responsive tissues is tightly regulated through biosynthesis, inactivation and transport. The mechanisms involved in regulating the expression of GA biosynthesis and inactivation genes in higher plants in response to developmental and environmental signals are active areas of research. The literature on this topic has been reviewed in detail (Hedden and Thomas 2012, Hedden 2016, Magome and Kamiya 2016) and will be summarized here, as well as highlighting some recent findings (see Fig.�5). CPS catalyzes the first committed step and is suggested to be the gateway to the GA-biosynthetic pathway with a role in developmental regulation (Silverstone et�al. 1997), but bioactive GA production is limited by later enzymes in the pathway, particularly GA20ox (Fleet et�al. 2003). Members of the 2-ODD gene families, which differ in their spatial and temporal expression patterns, are major sites of regulation. Transcription factors that specify spatial and temporal expression patterns of GA-biosynthetic and inactivation genes have been identified, including KNOX, MADS-box and bHLH proteins. A recent example is the MADS-box protein OsMADS57, which directly promotes expression of the inactivation genes *OsGA2ox3* and *OsEUI1* to limit internode elongation and panicle exsertion in rice (Chu et�al. 2019). Expression of *OsEUI1* was shown also to be promoted directly by the homeodomain-leucine zipper transcription factor HOX12 to regulate panicle exsertion (Gao et�al. 2016). In Arabidopsis, expression of an EUI1-like gene *EUI-LIKE P450 A1* (*ELA1*) is upregulated directly by LEAFY in floral primordia to suppress GA accumulation and promote flower formation (Yamaguchi et�al. 2014). In rice, mutation of the leucine zipper (HD-ZIP II) transcription factor *SMALL GRAIN AND DWARF 2* caused dwarfism, which was associated with reduced expression of *OsGA20ox1* and *OsGA20ox2* and increased expression of several *GA2ox* genes, although it is not known if the regulation is direct (Chen et�al. 2019).

**Fig 5:**
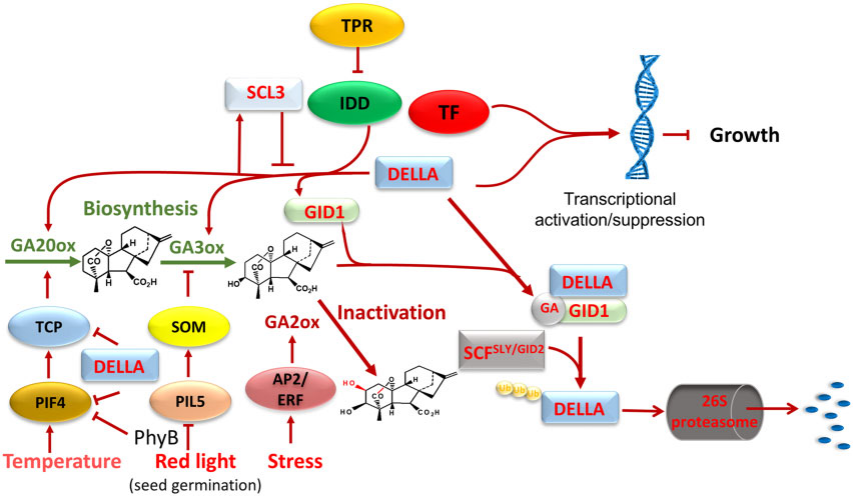
Regulation of GA biosynthesis and inactivation, highlighting the GA signal transduction pathway that enables GA homeostasis. The figure summarizes the data for Arabidopsis. GA signaling promotes the degradation of the DELLA transcriptional regulator, which in association with the transcription factor GAF1 upregulates the expression of genes encoding GA20ox and GA3ox, as well as the GA receptor GID1. Some pathways for the regulation of GA metabolism in response to environmental signals are also indicated.

Transcriptional regulation of GA metabolism genes via the GA signaling pathway provides a mechanism for GA homeostasis (Fig.�5): some *GA20ox* and *GA3ox* gene family members are downregulated by GA signaling whereas there is upregulation of *GA2ox* genes (see, e.g. Thomas et�al. 1999). Details of the GA signaling pathway are well established (Sun 2011, Nelson and Steber 2016). Briefly, binding of GA to its receptor GID1 results in a conformational change in the receptor that promotes its interaction with DELLA proteins, which then through association with the F-box component of an E3 ubiquitin ligase are targeted for degradation via the ubiquitin-proteasome pathway. DELLAs belong to the GRAS family of transcriptional regulators and uniquely contain an N-terminal sequence with conserved DELLA, LExLE and VHYNP domains that binds to the GA-GID1 complex to allow GA-induced degradation. DELLA function includes growth suppression, which is thus relieved by GA action. A major activity of DELLAs is to modify transcription in association with transcription factors (Davi�re and Achard 2016, Thomas et�al. 2016): this can be inhibitory through the sequestration of transcription factors so preventing their binding to gene promoters, or they can promote transcription by the sequestration of inhibitors or by acting as co-activators in partnership with transcription factors. This last mechanism is involved in the promotion of *GA20ox* and *GA3ox* expression by DELLAs to enable GA homeostasis through negative feedback regulation. In Arabidopsis, the DELLA protein GAI promotes transcription of *AtGA20ox2*, *AtGA3ox1* and the GA-receptor gene *AtGID1b* in association with the C2H2 zinc finger protein GAI-ASSOCIATED FACTOR1 (GAF1), also known as INDETERMINATE DOMAIN 2 (IDD2)(Fukazawa et�al. 2014). Activation of *AtGA20ox2* expression by GAF1 is suppressed by interaction with the WD-repeat protein TOPLESS-RELATED (TPR) such that expression is regulated by the balance between GAI and TPR. Fukazawa et�al. (2017) identified *cis* elements in the promoter of *AtGA20ox2* that are necessary for the binding of GAF1 and for feedback regulation indicating that, while other feedback mechanisms have been reported for some genes (reviewed in Hedden and Thomas 2012), GA regulation of *AtGA20ox2* expression is predominantly via GAF1. This transcription factor was also shown to specify the expression of *AtGA20ox2* in the shoot apex and root tip (Fukazawa et�al. 2017). A notable target of gene activation by IDD-DELLA encodes the non-DELLA GRAS protein SCARECROW-LIKE3 (SCL3) (Yoshida et�al. 2014). As SCL3 also interacts with IDDs, it attenuates its own expression by competing with DELLAs, as well as suppressing the expression of feedback regulated genes. The involvement of DELLAs in GA-induced upregulation of *GA2ox* genes is less clear and this process may involve other mechanisms (Livne et�al. 2015). While the 2-ODD genes are mainly implicated in homeostasis through GA metabolism, GA signaling has been reported to modify the expression of genes involved in earlier biosynthetic reactions, including downregulation of *KAO* expression in rice by the GA-responsive WUSCHEL-related homeobox factor OsWOX3A (Cho et�al. 2016). As discussed above, feedback regulation may also occur at the protein level (Lee and Zeevaart 2007) and, although it is more difficult to study, it warrants further investigation. In addition, allosteric regulation has been described for CPS, the activity of which is suppressed synergistically by Mg^2+^ and GGPP (Prisic and Peters 2007), and for OsGA2ox3, which is activated by its substrate GA_4_ (Takehara et�al. 2020), both mechanisms contributing to GA homeostasis.

Crosstalk between hormone signaling pathways is well established with DELLA proteins acting as a major hub. The evidence for other hormone signaling pathways targeting GA metabolism is conflicting (Ross et�al. 2011, Ross and Quittenden 2016), but there are examples for most hormones, which target primarily the GA 2-ODD genes (reviewed in Ross et�al. 2016). Notably, GA mediates growth stimulation by auxin, which promotes GA biosynthesis in a number of physiological contexts, including stem extension in response to auxin from the shoot apex (O’Neill and Ross 2002) and fruit growth induction by seed-derived auxin (Ozga and Reinecke 2003). It has been shown for several GA-biosynthetic genes that regulation by auxin occurs via the IAA/AUX ARF signaling pathway and is independent of DELLA (Frigerio et�al. 2006, O’Neill et�al. 2010).

A major function of GA is to mediate growth and developmental responses to environmental changes, which can cause rapid modification in GA concentration through altered metabolism. Environmental factors, including temperature, mechanical stimulation, abiotic and biotic stress and the duration, intensity and quality of light, have all been shown to affect GA biosynthesis and inactivation, acting primarily on the expression of the 2-ODD genes. In many cases, the transcription factors mediating these responses have been identified (reviewed in Hedden and Thomas 2012, Hedden 2016, Magome and Kamiya 2016). For example, stimulation of Arabidopsis seed germination by red light is associated with phytochrome-mediated degradation of the bHLH transcription factor PHYTOCHROME INTERACTING FACTOR-LIKE5 (PIL5), which restricts GA production by suppressing the expression of *AtGA3ox1* and *AtGA3ox2* and promoting *AtGA2ox2* expression in the dark via the intermediary of SOMNUS, a C3H-type zinc finger protein (Kim et�al. 2008). In the shade avoidance response, growth promotion under a low red/far-red light ratio was associated with enhanced expression of *GA20ox* genes in Arabidopsis petioles (Hisamatsu et�al. 2005), whereas in seedlings of the gymnosperm *Pinus tabuliformis*, under these conditions, *KAO* expression was strongly induced (Li et�al. 2020). Promotion of Arabidopsis seedling growth by transfer to higher temperature is associated with increased expression of *AtGA20ox1* and *AtGA3ox1* and decreased expression of *AtGA2ox1* in the hypocotyl (Stavang et�al. 2009). It was shown recently that in response to increased temperature, *AtGA20ox1* expression was directly upregulated by the class I TEOSINTE BRANCHED 1, CYCLOIDEA, PCF (TCP) transcription factors TCP14 and TCP15, which are induced by the temperature master regulator PHTOCHROME INTERACTING FACTOR4 (PIF4) (Ferrero et�al. 2019). As the function of both TCP (Davi�re et�al. 2014) and PIF4 (de Lucas et�al. 2008) is attenuated by interaction with DELLA proteins, the PIF4-TCP-GA signaling pathway is subject to complex feedback loops (Ferrero et�al. 2019). Growth suppression by abiotic stress through upregulation of *GA2ox* genes has been shown to be mediated by the stress-related APETALA2/ETHYLENE RESPONSE FACTOR (AP2/ERF)-type transcription factors (reviewed in Colebrook et�al. 2014). Recently, it was shown that microRNA regulation of an AP2 protein promoted stem elongation in barley, but the authors propose that AP2 acts through the jasmonate, rather than GA pathway to restrict internode elongation in this case (Patil et�al. 2019).

In common with other secondary metabolites, GA production by the fungus *F. fujikuroi* requires depletion of nitrogen sources, such as ammonium or glutamine (Tudzynski 2014). Through a complex interaction of the GATA transcription factors AreA and AreB, low nitrogen promotes the expression of six of the seven genes in the GA-biosynthetic cluster (Michielse et�al. 2014). Factors affecting the expression of the GA-biosynthetic operon in bacteria are less well understood, although in symbiotic rhizobia GA production is highly dependent on the developmental stage of the host plant (Mendez et�al. 2014).

## Concluding Remarks

Gibberellin biosynthesis is a mature field, which began in the late-1950s with work on GA_3_ biosynthesis in the fungus *F. fujikuroi*. Progress in the field was initially slow but accelerated with the development of sensitive methods for compound identification, the increasing availability of mutants and, more recently, of full genome sequences. These advances led first to the establishment of the metabolic pathways and then to the identification of the enzymes and finally of the relevant genes, in plants, fungi and recently bacteria. While genome sequences have proved extremely valuable for gene identification, it is important that gene function is not assumed from the sequence and is confirmed by biochemical means and/or through the use of mutants. The availability of genome sequences has prompted interest in the evolution of GA metabolism. Current information indicates that in plants GA biosynthesis evolved with vascularization, emphasizing the importance of GAs as a mobile signal. GA biosynthesis evolved also in fungi and bacteria that associate with plants enabling these organisms to function as pathogens or symbionts by modifying their hosts’ development and immunity. It is remarkable that the ability to produce these complex molecules has evolved three times in different kingdoms: plants, fungi and bacteria. There are reports of GAs occurring in algae, but as nonvascular land plants had not evolved the capability to synthesize GAs, it is unclear where algae fit in the evolutionary scheme. The reports need to be confirmed and the relevant enzymes identified.

Current work with flowering plants is focused on the mechanisms involved in regulating GA concentrations in response to developmental and environmental cues. While there is particular emphasis on the expression of GA biosynthesis and catabolism genes, it is also necessary to determine the sites of GA biosynthesis and action, ideally at the cellular level, and the mechanisms involved in linking them. Indeed, GA localization and movement is currently attracting considerable interest, e.g. associated with the identification of transporters. Locating the sites of GA accumulation at the cellular levels is an important goal that is being addressed through in vivo methods. Currently, for practical reasons, this has been restricted to locating the biologically active compounds, but determining the location of precursors can provide important information on how GA production is regulated. This will need to be addressed, and although the quantification of GAs and their precursors and catabolites at the cellular level by physicochemical methods is challenging, it is becoming more realistic with the increasing sensitivity of methods, such as UPLC–MS. The field has come a very long way in the last 60 years, but, through the implementation of technological advances, many more exciting discoveries can be anticipated.

## References

[pcaa092-B1] Aach H. , Bode H., Robinson D.G., Graebe J.E. (1997) *ent*-Kaurene synthase is located in proplastids of meristematic shoot tissues. Planta 202: 211–219.

[pcaa092-B2] Aach H. , B�se G., Graebe J.E. (1995) *ent*-Kaurene biosynthesis in a cell-free system from wheat (*Triticum aestivum* L.) seedlings and the localization of *ent*-kaurene synthetase in plastids of three species. Planta 197: 333–342.

[pcaa092-B3] Albone K.S. , Gaskin P., Macmillan J., Phinney B.O., Willis C.L. (1990) Biosynthetic origin of gibberellin A_3_ and gibberellin A_7_ in cell-free preparations from seeds of *Marah macrocarpus* and *Malus domestica*. Plant Physiol. 94: 132–142. 16667680 10.1104/pp.94.1.132 PMC1077201

[pcaa092-B4] Albone K.S. , Gaskin P., Macmillan J., Sponsel V.M. (1984) Identification and localization of gibberellins in maturing seeds of the cucurbit *Sechium edule*, and a comparison between this cucurbit and the legume *Phaseolus coccineus*. Planta 162: 560–565. 24253274 10.1007/BF00399923

[pcaa092-B5] Appleford N.E.J. , Evans D.J., Lenton J.R., Gaskin P., Croker S.J., Devos K.M., et al (2006) Function and transcript analysis of gibberellin-biosynthetic enzymes in wheat. Planta 223: 568–582. 16160850 10.1007/s00425-005-0104-0

[pcaa092-B6] Appleford N.E.J. , Lenton J.R. (1997) Hormonal regulation of α-amylase gene expression in germinating wheat (*Triticum aestivum*) grains. Physiol. Plant. 100: 534–542.

[pcaa092-B7] Beale M.H. , Bearder J.R., Down G.H., Hutchison M., Macmillan J., Phinney B.O. (1982) The biosynthesis of kaurenolide diterpenoids by *Gibberella fujikuro*I. Phytochemistry 21: 1279–1287.

[pcaa092-B8] Bearder J.R. , MacMillan J., Phinney B.O. (1975) Fungal products. Part XIV. Metabolic pathways from *ent*-kaurenoic acid to fungal gibberellins in mutant B1-41a of *Gibberella fujikuroi*. J. Chem. Soc. Perkin Trans. 1 1: 721–726.

[pcaa092-B9] Bearder J.R. , MacMillan J., Phinney B.O. (1976) Origin of oxygen atoms in lactone bridge of C_19_-gibberellins. J. Chem. Soc, Chem. Commun. 834–835.

[pcaa092-B10] Beck G. , Coman D., Herren E., Ruiz-Sola M., Rodriguez-Concepcion M., Gruissem W., et al (2013) Characterization of the GGPP synthase gene family in *Arabidopsis thaliana*. Plant Mol. Biol. 82: 393–416. 23729351 10.1007/s11103-013-0070-z

[pcaa092-B11] Bhattacharya A. , Kourmpetli S., Ward D.A., Thomas S.G., Gong F., Powers S.J., et al (2012) Characterization of the fungal gibberellin desaturase as a 2-oxoglutarate-dependent dioxygenase and its utilization for enhancing plant growth. Plant Physiol. 160: 837–845. 22911627 10.1104/pp.112.201756 PMC3461559

[pcaa092-B12] Binenbaum J. , Weinstain R., Shani E. (2018) Gibberellin localization and transport in plants. Trends Plant. Sci 23: 410–421. 29530380 10.1016/j.tplants.2018.02.005

[pcaa092-B13] Birnbaum K. , Shasha D.E., Wang J.Y., Jung J.W., Lambert G.M., Galbraith D.W., et al (2003) A gene expression map of the Arabidopsis root. Science 302: 1956–1960. 14671301 10.1126/science.1090022

[pcaa092-B14] Block M.A. , Jouhet J. (2015) Lipid trafficking at endoplasmic reticulum-chloroplast membrane contact sites. Curr. Opin. Cell Biol. 35: 21–29. 25868077 10.1016/j.ceb.2015.03.004

[pcaa092-B15] Bolduc N. , Hake S. (2009) The maize transcription factor KNOTTED1 directly regulates the gibberellin catabolism gene *ga2ox1*. Plant Cell 21: 1647–1658. 19567707 10.1105/tpc.109.068221 PMC2714931

[pcaa092-B16] B�mke C. , Rojas M.C., Gong F., Hedden P., Tudzynski B. (2008) Isolation and characterization of the gibberellin biosynthetic gene cluster in *Sphaceloma manihoticola*. Appl. Environ. Microbiol. 74: 5325–5339. 18567680 10.1128/AEM.00694-08 PMC2546651

[pcaa092-B17] Brautigam A. , Weber A.P.M. (2009) Proteomic analysis of the proplastid envelope membrane provides novel insights into small molecule and protein transport across proplastid membranes. Mol. Plant 2: 1247–1261. 19995728 10.1093/mp/ssp070

[pcaa092-B18] Castellaro S.J. , Dolan S.C., Hedden P., Gaskin P., Macmillan J. (1990) Stereochemistry of the metabolic steps from kaurenoic acids to kaurenolides and gibberellins. Phytochemistry 29: 1833–1839.

[pcaa092-B19] Chen W.W. , Cheng Z.J., Liu L.L., Wang M., You X.M., Wang J. (2019) Small Grain and Dwarf 2, encoding an HD-Zip II family transcription factor, regulates plant development by modulating gibberellin biosynthesis in rice. Plant Sci. 288: 110208. 31521223 10.1016/j.plantsci.2019.110208

[pcaa092-B20] Chen X. , Tian X.J., Xue L., Zhang X.H., Yang S.H., Traw M.B., et al (2019) CRISPR-based assessment of gene specialization in the gibberellin metabolic pathway in rice. Plant Physiol. 180: 2091–2105. 31160507 10.1104/pp.19.00328 PMC6670093

[pcaa092-B21] Cho S.H. , Kang K., Lee S.H., Lee I.J., Paek N.C. (2016) OsWOX3A is involved in negative feedback regulation of the gibberellic acid biosynthetic pathway in rice (*Oryza sativa*). J. Exp. Bot. 67: 1677–1687. 26767749 10.1093/jxb/erv559 PMC4783357

[pcaa092-B22] Chu Y.L. , Xu N., Wu Q., Yu B., Li X.X., Chen R.R., et al (2019) Rice transcription factor OsMADS57 regulates plant height by modulating gibberellin catabolism. Rice 12: 38. 31139953 10.1186/s12284-019-0298-6 PMC6538746

[pcaa092-B23] Colebrook E.H. , Thomas S.G., Phillips A.L., Hedden P. (2014) The role of gibberellin signalling in plant responses to abiotic stress. J. Exp. Biol. 217: 67–75. 24353205 10.1242/jeb.089938

[pcaa092-B24] Crozier A. , Kuo C.C., Durley R.C., Pharis R.P. (1970) Biological activities of 26 gibberellins in nine plant bioassays. Can. J. Bot. 48: 867–877.

[pcaa092-B25] Croker S.J. , Gaskin P., Beale M.H., Lenton J.R. (1995) *ent*-3β-Hydroxykaur-16-ene and *ent*-17-hydroxykaur-15-ene in paclobutrazol-treated wheat seedlings. Phytochemistry 39: 11–14.

[pcaa092-B26] Curtis P.J. , Cross B.E. (1954) Gibberellic acid—a new metabolite from the culture filtrates of *Gibberella fujikuroi*. Chem. Ind 1066.

[pcaa092-B27] Davi�re J.M. , Achard P. (2016) A pivotal role of DELLAs in regulating multiple hormone signals. Mol. Plant 9: 10–20. 26415696 10.1016/j.molp.2015.09.011

[pcaa092-B28] Davi�re J.M. , Wild M., Regnault T., Baumberger N., Eisler H., Genschik P., et al (2014) Class I TCP-DELLA interactions in inflorescence shoot apex determine plant height. Curr. Biol. 24: 1923–1928. 25127215 10.1016/j.cub.2014.07.012

[pcaa092-B29] de Lucas M. , Daviere J.M., Rodriguez-Falcon M., Pontin M., Iglesias-Pedraz J.M., Lorrain S., et al (2008) A molecular framework for light and gibberellin control of cell elongation. Nature 451: 480–484. 18216857 10.1038/nature06520

[pcaa092-B30] Ding Y. , Murphy K.M., Poretsky E., Mafu S., Yang B., Char S.N., et al (2019) Multiple genes recruited from hormone pathways partition maize diterpenoid defences. Nat. Plants 5: 1043–1056. 31527844 10.1038/s41477-019-0509-6

[pcaa092-B31] Dugardeyn J. , Vandenbussche F., Van Der Straeten D. (2008) To grow or not to grow: what can we learn on ethylene-gibberellin cross-talk by in silico gene expression analysis? J. Exp. Bot. 59: 1–16. 18212030 10.1093/jxb/erm349

[pcaa092-B32] Eriksson S. , Bohlenius H., Moritz T., Nilsson O. (2006) GA_4_ is the active gibberellin in the regulation of *LEAFY* transcription and Arabidopsis floral initiation. Plant Cell 18: 2172–2181. 16920780 10.1105/tpc.106.042317 PMC1560906

[pcaa092-B33] Ferrero L.V. , Viola I.L., Ariel F.D., Gonzalez D.H. (2019) Class I TCP transcription factors target the gibberellin biosynthesis gene GA20OX1 and the growth-promoting genes *HBI1* and *PRE6* during thermomorphogenic growth in *Arabidopsis*. Plant Cell Physiol. 60: 1633–1645. 31292642 10.1093/pcp/pcz137

[pcaa092-B34] Fixen K.R. , Thomas S.C., Tong C.B.S. (2012) Blue light inhibition of tuberization in a day-neutral potato. J. Plant Growth Regul. 31: 342–350.

[pcaa092-B35] Fleet C.M. , Yamaguchi S., Hanada A., Kawaide H., David C.J., Kamiya Y., et al (2003) Overexpression of AtCPS and AtKS in Arabidopsis confers increased *ent*-kaurene production but no increase in bioactive gibberellins. Plant Physiol. 132: 830–839. 12805613 10.1104/pp.103.021725 PMC167023

[pcaa092-B36] Flugge U.I. , Gao W. (2005) Transport of isoprenoid intermediates across chloroplast envelope membranes. Plant Biol. 7: 91–97. 15666208 10.1055/s-2004-830446

[pcaa092-B37] Ford B.A. , Foo E., Sharwood R., Karafiatova M., Vrana J., MacMillan C., et al (2018) Rht18 semidwarfism in wheat is due to increased GA 2-oxidaseA9 expression and reduced GA content. Plant Physiol. 177: 168–180. 29545269 10.1104/pp.18.00023 PMC5933146

[pcaa092-B38] Frigerio M. , Alabadi D., Perez-Gomez J., Garcia-Carcel L., Phillips A.L., Hedden P., et al (2006) Transcriptional regulation of gibberellin metabolism genes by auxin signaling in arabidopsis. Plant Physiol. 142: 553–563. 16905669 10.1104/pp.106.084871 PMC1586059

[pcaa092-B39] Frisse A. , Pimenta M.J., Lange T. (2003) Expression studies of gibberellin oxidases in developing pumpkin seeds. Plant Physiol. 131: 1220–1227. 12644672 10.1104/pp.015206 PMC166882

[pcaa092-B40] Fu J.Y. , Ren F., Lu X., Mao H.J., Xu M.M., Degenhardt J., et al (2016) A tandem array of *ent*-kaurene synthases in maize with roles in gibberellin and more specialized metabolism. Plant Physiol. 170: 742–751. 26620527 10.1104/pp.15.01727 PMC4734586

[pcaa092-B41] Fujioka S. , Yamane H., Spray C.R., Phinney B.O., Gaskin P., Macmillan J., et al (1990) Gibberellin A_3_ is biosynthesized from gibberellin A_20_ via gibberellin A_5_ in shoots of *Zea mays* L. Plant Physiol. 94: 127–131. 16667678 10.1104/pp.94.1.127 PMC1077200

[pcaa092-B42] Fukazawa J. , Mori M., Watanabe S., Miyamoto C., Ito T., Takahashi Y. (2017) DELLA-GAF1 complex is a main component in gibberellin feedback regulation of GA 20-oxidase2. Plant Physiol. 175: 1395–1406. 28916594 10.1104/pp.17.00282 PMC5664458

[pcaa092-B43] Fukazawa J. , Teramura H., Murakoshi S., Nasuno K., Nishida N., Ito T., et al (2014) DELLAs function as coactivators of GAI-ASSOCIATED FACTOR1 in regulation of gibberellin homeostasis and signaling in Arabidopsis. Plant Cell 26: 2920–2938. 25035403 10.1105/tpc.114.125690 PMC4145123

[pcaa092-B44] Gao S.P. , Fang J., Xu F., Wang W., Chu C.C. (2016) Rice HOX12 regulates panicle exsertion by directly modulating the expression of ELONGATED UPPERMOST INTERNODE1. Plant Cell 28: 680–695. 26977084 10.1105/tpc.15.01021 PMC4826014

[pcaa092-B45] Gaskin P. , Kirkwood P.S., Macmillan J. (1981) Partial synthesis of *ent*-13-hydroxy-2-oxo-20-norgibberella-1(10),16-diene-7,19-dioic acid, a catabolite of gibberellin A_29_, and of related-compounds. J. Chem. Soc. Perkin Trans. 1: 1083–1091.

[pcaa092-B46] Gilmour S.J. , Zeevaart J.A.D., Schwenen L., Graebe J.E. (1986) Gibberellin metabolism in cell-free-extracts from spinach leaves in relation to photoperiod. Plant Physiol. 82: 190–195. 16664991 10.1104/pp.82.1.190 PMC1056088

[pcaa092-B47] Graebe J.E. , Hedden P., MacMillan J. (1975) Ring contraction step in gibberellin biosynthesis. J. Chem. Soc. Chem. Commun. 1975: 161–162.

[pcaa092-B48] Han F.M. , Zhu B.G. (2011) Evolutionary analysis of three gibberellin oxidase genes in rice, Arabidopsis, and soybean. Gene 473: 23–35. 21056641 10.1016/j.gene.2010.10.010

[pcaa092-B49] Hayashi K. , Kawaide H., Notomi M., Sakigi Y., Matsuo A., Nozaki H. (2006) Identification and functional analysis of bifunctional *ent*-kaurene synthase from the moss *Physcomitrella patens*. FEBS Lett. 580: 6175–6181. 17064690 10.1016/j.febslet.2006.10.018

[pcaa092-B50] He J. , Chen Q., Xin P., Yuan J., Ma Y., Wang X., et al (2019) CYP72A enzymes catalyse 13-hydrolyzation of gibberellins. Nat. Plants 5: 1057–1065. 31527846 10.1038/s41477-019-0511-z PMC7194175

[pcaa092-B51] Hedden P. (2016) Gibberellin biosynthesis in higher plants. Annu. Plant Rev. 49: 37–72.

[pcaa092-B52] Hedden P. , Graebe J.E. (1981) Kaurenolide biosynthesis in a cell-free system from *Cucurbita maxima* seeds. Phytochemistry 20: 1011–1015.

[pcaa092-B53] Hedden P. , Sponsel V. (2015) A century of gibberellin research. J. Plant Growth Regul. 34: 740–760. 26523085 10.1007/s00344-015-9546-1 PMC4622167

[pcaa092-B54] Hedden P. , Thomas S.G. (2012) Gibberellin biosynthesis and its regulation. Biochem. J 444: 11–25. 22533671 10.1042/BJ20120245

[pcaa092-B55] Helliwell C.A. , Chandler P.M., Poole A., Dennis E.S., Peacock W.J. (2001) The CYP88A cytochrome P450, *ent*-kaurenoic acid oxidase, catalyzes three steps of the gibberellin biosynthesis pathway. Proc. Natl. Acad. Sci. USA 98: 2065–2070. 11172076 10.1073/pnas.041588998 PMC29382

[pcaa092-B56] Helliwell C.A. , Poole A., Peacock W.J., Dennis E.S. (1999) Arabidopsis *ent*-kaurene oxidase catalyzes three steps of gibberellin biosynthesis. Plant Physiol. 119: 507–510. 9952446 10.1104/pp.119.2.507 PMC32127

[pcaa092-B57] Helliwell C.A. , Sullivan J.A., Mould R.M., Gray J.C., Peacock W.J., Dennis E.S. (2001) A plastid envelope location of *Arabidopsis ent*-kaurene oxidase links the plastid and endoplasmic reticulum steps of the gibberellin biosynthesis pathway. Plant J. 28: 201–208. 11722763 10.1046/j.1365-313x.2001.01150.x

[pcaa092-B58] Hirano K. , Aya K., Hobo T., Sakakibara H., Kojima M., Shim R.A., et al (2008) Comprehensive transcriptome analysis of phytohormone biosynthesis and signaling genes in microspore/pollen and tapetum of rice. Plant Cell Physiol. 49: 1429–1450. 18718932 10.1093/pcp/pcn123 PMC2566925

[pcaa092-B59] Hisamatsu T. , King R.W., Helliwell C.A., Koshioka M. (2005) The involvement of gibberellin 20-oxidase genes in phytochrome-regulated petiole elongation of Arabidopsis. Plant Physiol. 138: 1106–1116. 15923331 10.1104/pp.104.059055 PMC1150424

[pcaa092-B60] Hu J.H. , Mitchum M.G., Barnaby N., Ayele B.T., Ogawa M., Nam E., et al (2008) Potential sites of bioactive gibberellin production during reproductive growth in Arabidopsis. Plant Cell 20: 320–336. 18310462 10.1105/tpc.107.057752 PMC2276448

[pcaa092-B61] Huang Y. , Wang X., Ge S., Rao G.Y. (2015) Divergence and adaptive evolution of the gibberellin oxidase genes in plants. BMC Evol. Biol. 15: 207. 26416509 10.1186/s12862-015-0490-2 PMC4587577

[pcaa092-B62] Itoh H. , Tatsumi T., Sakamoto T., Otomo K., Toyomasu T., Kitano H., et al (2004) A rice semi-dwarf gene, *Tan-Ginbozu* (*D35*), encodes the gibberellin biosynthesis enzyme, *ent*-kaurene oxidase. Plant Mol. Biol. 54: 533–547. 15316288 10.1023/B:PLAN.0000038261.21060.47

[pcaa092-B63] Itoh H. , Ueguchi-Tanaka M., Sentoku N., Kitano H., Matsuoka M., Kobayashi M. (2001) Cloning and functional analysis of two gibberellin 3β-hydroxylase genes that are differently expressed during the growth of rice. Proc. Natl. Acad. Sci. USA 98: 8909–8914. 11438692 10.1073/pnas.141239398 PMC37534

[pcaa092-B64] Jacob D. , Brian J. (2020) The short and intricate life of the suspensor. Physiol. Plant. 169: 110–121. 31808953 10.1111/ppl.13057

[pcaa092-B65] Jasinski S. , Piazza P., Craft J., Hay A., Woolley L., Rieu I., et al (2005) KNOX action in *Arabidopsis* is mediated by coordinate regulation of cytokinin and gibberellin activities. Curr. Biol. 15: 1560–1565. 16139211 10.1016/j.cub.2005.07.023

[pcaa092-B66] Kamiya Y. , Graebe J.E. (1983) The biosynthesis of all major pea gibberellins in a cell-free system from *Pisum sativum*. Phytochemistry 22: 681–689.

[pcaa092-B67] Kamiya Y. , Takahashi N., Graebe J.E. (1986) The loss of carbon-20 in C_19_-gibberellin biosynthesis in a cell-free system from *Pisum sativum* L. Planta 169: 524–528. 24232760 10.1007/BF00392102

[pcaa092-B68] Kaneko M. , Itoh H., Inukai Y., Sakamoto T., Ueguchi-Tanaka M., Ashikari M., et al (2003) Where do gibberellin biosynthesis and gibberellin signaling occur in rice plants? Plant J. 35: 104–115. 12834406 10.1046/j.1365-313x.2003.01780.x

[pcaa092-B69] Kasahara H. , Hanada A., Kuzuyama T., Takagi M., Kamiya Y., Yamaguchi S. (2002) Contribution of the mevalonate and methylerythritol phosphate pathways to the biosynthesis of gibberellins in *Arabidopsis*. J. Biol. Chem. 277: 45188–45194. 12228237 10.1074/jbc.M208659200

[pcaa092-B70] Katsarou K. , Wu Y., Zhang R.X., Bonar N., Morris J., Hedley P.E., et al (2016) Insight on genes affecting tuber development in potato upon potato spindle tuber viroid (PSTVd) infection. PLoS One 11: e0150711. 26937634 10.1371/journal.pone.0150711 PMC4777548

[pcaa092-B71] Kawai Y. , Ono E., Mizutani M. (2014) Evolution and diversity of the 2-oxoglutarate-dependent dioxygenase superfamily in plants. Plant J. 78: 328–343. 24547750 10.1111/tpj.12479

[pcaa092-B72] Kawaide H. (2006) Biochemical and molecular analyses of gibberellin biosynthesis in fungi. Biosci. Biotechnol. Biochem. 70: 583–590. 16556972 10.1271/bbb.70.583

[pcaa092-B73] Keeling C.I. , Dullat H.K., Yuen M., Ralph S.G., Jancsik S., Bohlmann J. (2010) Identification and functional characterization of monofunctional *ent*-copalyl diphosphate and ent-kaurene synthases in white spruce reveal different patterns for diterpene synthase evolution for primary and secondary metabolism in gymnosperms. Plant Physiol. 152: 1197–1208. 20044448 10.1104/pp.109.151456 PMC2832265

[pcaa092-B74] Kell D.B. (2015) What would be the observable consequences if phospholipid bilayer diffusion of drugs into cells is negligible? Trends Pharmacol. Sci. 36: 15–21. 25458537 10.1016/j.tips.2014.10.005

[pcaa092-B75] Kim D.H. , Yamaguchi S., Lim S., Oh E., Park J., Hanada A., et al (2008) SOMNUS, a CCCH-type zinc finger protein in Arabidopsis, negatively regulates light-dependent seed germination downstream of PIL5. Plant Cell 20: 1260–1277. 18487351 10.1105/tpc.108.058859 PMC2438461

[pcaa092-B76] King R.W. , Mander L.N., Asp T., MacMillan C.P., Blundell C.A., Evans L.T. (2008) Selective deactivation of gibberellins below the shoot apex is critical to flowering but not to stem elongation of *Lolium*. Mol. Plant 1: 295–307. 19825541 10.1093/mp/ssm030

[pcaa092-B77] King R.W. , Moritz T., Evans L.T., Junttila O., Herlt A.J. (2001) Long-day induction of flowering in *Lolium temulentum* involves sequential increases in specific gibberellins at the shoot apex. Plant Physiol. 127: 624–632. 11598236 PMC125097

[pcaa092-B78] Kobayashi M. , Yamaguchi I., Murofushi N., Ota Y., Takahashi N. (1988) Fluctuation and localization of endogenous gibberellins in rice. Agric. Biol. Chem. 52: 1189–1194.

[pcaa092-B79] Koksal M. , Potter K., Peters R.J., Christianson D.W. (2014) 1.55 angstrom-resolution structure of ent-copalyl diphosphate synthase and exploration of general acid function by site-directed mutagenesis. Biochim. Biophys. Acta Gen. Subjects 1840: 184–190. 10.1016/j.bbagen.2013.09.004 PMC3859867 24036329

[pcaa092-B80] Kramer E.M. (2006) How far can a molecule of weak acid travel in the apoplast or xylem? Plant Physiol. 141: 1233–1236. 16896235 10.1104/pp.106.083790 PMC1533949

[pcaa092-B81] Lacombe B. , Achard P. (2016) Long-distance transport of phytohormones through the plant vascular system. Curr. Opin. Plant Biol. 34: 1–8. 27340874 10.1016/j.pbi.2016.06.007

[pcaa092-B82] Lange M.J.P. , Lange T. (2015) Touch-induced changes in *Arabidopsis* morphology dependent on gibberellin breakdown. Nat. Plants 1: 14025. 27246879 10.1038/nplants.2014.25

[pcaa092-B83] Lange M.J.P. , Lange T. (2016) Ovary-derived precursor gibberellin A_9_ is essential for female flower development in cucumber. Development 143: 4425–4429. 27789625 10.1242/dev.135947

[pcaa092-B84] Lange M.J.P. , Liebrandt A., Arnold L., Chmielewska S.M., Felsberger A., Freier E., et al (2013) Functional characterization of gibberellin oxidases from cucumber, *Cucumis sativus* L. Phytochemistry 90: 62–69. 23507362 10.1016/j.phytochem.2013.02.006

[pcaa092-B85] Lange T. , Hedden P., Graebe J.E. (1994) Expression cloning of a gibberellin 20-oxidase, a multifunctional enzyme involved in gibberellin biosynthesis. Proc. Natl. Acad. Sci. USA 91: 8552–8556. 8078921 10.1073/pnas.91.18.8552 PMC44644

[pcaa092-B86] Lange T. , Kappler J., Fischer A., Frisse A., Padeffke T., Schmidtke S., et al (2005) Gibberellin biosynthesis in developing pumpkin seedlings. Plant Physiol. 139: 213–223. 16126862 10.1104/pp.105.064162 PMC1203371

[pcaa092-B87] Lange T. , Schweimer A., Ward D.A., Hedden P., Graebe J.E. (1994) Separation and characterization of three 2-oxoglutarate-dependent dioxygenases from *Cucurbita maxima* L. endosperm involved in gibberellin biosynthesis. Planta 195: 98–107.

[pcaa092-B88] Lee D.J. , Zeevaart J.A.D. (2007) Regulation of gibberellin 20-oxidase1 expression in spinach by photoperiod. Planta 226: 35–44. 17216482 10.1007/s00425-006-0463-1

[pcaa092-B89] Lemke C. , Potter K.C., Schulte S., Peters R.J. (2019) Conserved bases for the initial cyclase in gibberellin biosynthesis: from bacteria to plants. Biochem. J. 476: 2607–2621. 31484677 10.1042/BCJ20190479 PMC7244331

[pcaa092-B90] Lester D.R. , Ross J.J., Smith J.J., Elliott R.C., Reid J.B. (1999) Gibberellin 2-oxidation and the *SLN* gene of *Pisum sativum*. Plant J. 19: 65–73. 10417727 10.1046/j.1365-313x.1999.00501.x

[pcaa092-B91] Li C. , Zheng L.L., Wang X.N., Hu Z.B., Zheng Y., Chen Q.H., et al (2019) Comprehensive expression analysis of Arabidopsis GA2-oxidase genes and their functional insights. Plant Sci. 285: 1–13. 31203874 10.1016/j.plantsci.2019.04.023

[pcaa092-B92] Li W. , Liu S.W., Ma J.J., Liu H.M., Han F.X., Li Y., et al (2020) Gibberellin signaling is required for far-red light-induced shoot elongation in *Pinus tabuliformis* seedlings. Plant Physiol. 182: 658–668. 31659126 10.1104/pp.19.00954 PMC6945873

[pcaa092-B93] Liang M.Z. , Deng L.X., Liu J.F., He A.N., Chen L.B. (2008) Interaction between the *eui* gene and thermo-sensitive genic male sterility in rice. Euphytica 164: 637–643.

[pcaa092-B94] Liu H. , Guo S.Y., Lu M.H., Zhang Y., Li J.H., Wang W., et al (2019) Biosynthesis of DHGA_12_ and its roles in *Arabidopsis* seedling establishment. Nat. Commun. 10: 1768. 30992454 10.1038/s41467-019-09467-5 PMC6467921

[pcaa092-B95] Liu W.T. , Feng X.X., Zheng Y.Y., Huang C.H., Nakano C., Hoshino T., et al (2014) Structure, function and inhibition of ent-kaurene synthase from *Bradyrhizobium japonicum*. Sci. Rep. 4: 6214. 25269599 10.1038/srep06214 PMC4180811

[pcaa092-B96] Livne S. , Lor V.S., Nir I., Eliaz N., Aharoni A., Olszewski N.E., et al (2015) Uncovering DELLA-independent gibberellin responses by characterizing new tomato procera mutants. Plant Cell 27: 1579–1594. 26036254 10.1105/tpc.114.132795 PMC4498196

[pcaa092-B97] Lu X. , Hershey D.M., Wang L., Bogdanove A.J., Peters R.J. (2015) An *ent*-kaurene-derived diterpenoid virulence factor from *Xanthomonas oryzae* pv. oryzicola. New Phytol. 206: 295–302. 25406717 10.1111/nph.13187

[pcaa092-B98] MacMillan J. (2001) Occurrence of gibberellins in vascular plants, fungi, and bacteria. J. Plant Growth Regul. 20: 387–442. 11986764 10.1007/s003440010038

[pcaa092-B99] MacMillan J. , Suter P.J. (1958) The occurrence of gibberellin A_1_ in higher plants—isolation from the seed of runner bean (*Phaseolus multiflorus*). Naturwissenschaften 45: 46–46.

[pcaa092-B100] MacMillan J. , Takahashi N. (1968) Proposed procedure for allocation of trivial names to gibberellins. Nature 217: 170–171. 5638147 10.1038/217170a0

[pcaa092-B101] Magome H. , Kamiya Y. (2016) Inactivation processes. Annu. Plant Rev. 49: 73–94.

[pcaa092-B102] Magome H. , Nomura T., Hanada A., Takeda-Kamiya N., Ohnishi T., Shinma Y., et al (2013) *CYP714B1* and *CYP714B2* encode gibberellin 13-oxidases that reduce gibberellin activity in rice. Proc. Natl. Acad. Sci. USA 110: 1947–1952. 23319637 10.1073/pnas.1215788110 PMC3562828

[pcaa092-B103] Malonek S. , Bomke C., Bornberg-Bauer E., Rojas M.C., Hedden P., Hopkins P., et al (2005) Distribution of gibberellin biosynthetic genes and gibberellin production in the *Gibberella fujikuroi* species complex. Phytochemistry 66: 1296–1311. 15925394 10.1016/j.phytochem.2005.04.012

[pcaa092-B104] Mann F.M. , Prisic S., Davenport E.K., Determan M.K., Coates R.M., Peters R.J. (2010) A single residue switch for Mg^2+^-dependent inhibition characterizes plant class II diterpene cyclases from primary and secondary metabolism. J. Biol. Chem. 285: 20558–20563. 20430888 10.1074/jbc.M110.123307 PMC2898348

[pcaa092-B105] McAdam E.L. , Reid J.B., Foo E. (2018) Gibberellins promote nodule organogenesis but inhibit the infection stages of nodulation. J. Exp. Bot. 69: 2117–2130. 29432555 10.1093/jxb/ery046 PMC6018947

[pcaa092-B106] Mehrshahi P. , Stefano G., Andaloro J.M., Brandizzi F., Froehlich J.E., DellaPenna D. (2013) Transorganellar complementation redefines the biochemical continuity of endoplasmic reticulum and chloroplasts. Proc. Natl. Acad. Sci. USA 110: 12126–12131. 23818635 10.1073/pnas.1306331110 PMC3718160

[pcaa092-B107] Mendez C. , Baginsky C., Hedden P., Gong F., Caru M., Rojas M.C. (2014) Gibberellin oxidase activities in *Bradyrhizobium japonicum* bacteroids. Phytochemistry 98: 101–109. 24378220 10.1016/j.phytochem.2013.11.013

[pcaa092-B108] Michielse C.B. , Pfannmuller A., Macios M., Rengers P., Dzikowska A., Tudzynski B. (2014) The interplay between the GATA transcription factors AreA, the global nitrogen regulator and AreB in *Fusarium fujikuroi*. Mol. Microbiol. 91: 472–493. 24286256 10.1111/mmi.12472

[pcaa092-B109] Miyazaki S. , Hara M., Ito S., Tanaka K., Asami T., Hayashi K., et al (2018) An ancestral gibberellin in a moss *Physcomitrella patens*. Mol. Plant 11: 1097–1100. 29571905 10.1016/j.molp.2018.03.010

[pcaa092-B110] Morrone D. , Chambers J., Lowry L., Kim G., Anterola A., Bender K., et al (2009) Gibberellin biosynthesis in bacteria: separate *ent*-copalyl diphosphate and *ent*-kaurene synthases in *Bradyrhizobium japonicum*. FEBS Lett. 583: 475–480. 19121310 10.1016/j.febslet.2008.12.052

[pcaa092-B111] Murphy K.M. , Ma L.T., Ding Y.Z., Schmelz E.A., Zerbe P. (2018) Functional characterization of two class II diterpene synthases indicates additional specialized diterpenoid pathways in maize (*Zea mays*). Front. Plant Sci. 9: 1542. 30405674 10.3389/fpls.2018.01542 PMC6206430

[pcaa092-B112] Myllyla R. , Tuderman L., Kivirikko K.I. (1977) Mechanism of prolyl hydroxylase reaction. 2. Kinetic analysis of reaction sequence. Eur. J. Biochem. 80: 349–357. 200425 10.1111/j.1432-1033.1977.tb11889.x

[pcaa092-B113] Nagel R. , Peters R.J. (2017) ^18^O_2_ labeling experiments illuminate the oxidation of *ent*-kaurene in bacterial gibberellin biosynthesis. Org. Biomol. Chem. 15: 7566–7571. 28858359 10.1039/c7ob01819c

[pcaa092-B114] Nagel R. , Peters R.J. (2017) Investigating the phylogenetic range of gibberellin biosynthesis in bacteria. Mol. Plant Microbe Interct. 30: 343–349. 10.1094/MPMI-01-17-0001-R PMC5505637 28425831

[pcaa092-B115] Nagel R. , Peters R.J. (2018) Diverging mechanisms: cytochrome P450-catalyzed demethylation and γ-lactone formation in bacterial gibberellin biosynthesis. Angew. Chem. Int. Ed. 57: 6082–6085. 10.1002/anie.201713403 PMC6020689 29517843

[pcaa092-B116] Nagel R. , Turrini P.C.G., Nett R.S., Leach J.E., Verdier V., Van Sluys M.-A., et al (2017) An operon for production of bioactive gibberellin A_4_ phytohormone with wide distribution in the bacterial rice leaf streak pathogen *Xanthomonas oryzae* pv. *oryzicola*. New Phytol. 214: 1260–1266. 28134995 10.1111/nph.14441 PMC5388578

[pcaa092-B117] Navarro L. , Bari R., Achard P., Lison P., Nemri A., Harberd N.P., et al (2008) DELLAs control plant immune responses by modulating the balance and salicylic acid signaling. Curr. Biol. 18: 650–655. 18450451 10.1016/j.cub.2008.03.060

[pcaa092-B118] Nelson S.K. , Steber C.M. (2016) Gibberellin hormone signal perception: down-regulating DELLA repressors of plant growth and development. Annu. Plant Rev. 49: 153–158.

[pcaa092-B119] Nett R.S. , Contreras T., Peters R.J. (2017) Characterization of CYP115 as a gibberellin 3-oxidase indicates that certain rhizobia can produce bioactive gibberellin A_4_. ACS Chem. Biol. 12: 912–917. 28199080 10.1021/acschembio.6b01038 PMC5404427

[pcaa092-B120] Nett R.S. , Dickschat J.S., Peters R.J. (2016) Labeling studies clarify the committed step in bacterial gibberellin biosynthesis. Org. Lett. 18: 5974–5977. 27934361 10.1021/acs.orglett.6b02569 PMC5139915

[pcaa092-B121] Nett R.S. , Montanares M., Marcassa A., Lu X., Nagel R., Charles T.C., et al (2017) Elucidation of gibberellin biosynthesis in bacteria reveals convergent evolution. Nat. Chem. Biol. 13: 69–74. 27842068 10.1038/nchembio.2232 PMC5193102

[pcaa092-B122] Nishijima T. , Katsura N. (1989) A modified micro-drop bioassay using dwarf rice for detection of femtomol quantities of gibberellins. Plant Cell Physiol. 30: 623–627.

[pcaa092-B123] Niu S.H. , Yuan L., Zhang Y.C., Chen X.Y., Li W. (2014) Isolation and expression profiles of gibberellin metabolism genes in developing male and female cones of *Pinus tabuliformis*. Funct. Integr. Genomics 14: 697–705. 25091154 10.1007/s10142-014-0387-y

[pcaa092-B124] Nomura T. , Magome H., Hanada A., Takeda-Kamiya N., Mander L.N., Kamiya Y., et al (2013) Functional analysis of *Arabidopsis* CYP714A1 and CYP714A2 reveals that they are distinct gibberellin modification enzymes. Plant Cell Physiol. 54: 1837–1851. 24009336 10.1093/pcp/pct125

[pcaa092-B125] O’Neill D.P. , Davidson S.E., Clarke V.C., Yamauchi Y., Yamaguchi S., Kamiya Y., et al (2010) Regulation of the gibberellin pathway by auxin and DELLA proteins. Planta 232: 1141–1149. 20706734 10.1007/s00425-010-1248-0

[pcaa092-B126] O’Neill D.P. , Ross J.J. (2002) Auxin regulation of the gibberellin pathway in pea. Plant Physiol. 130: 1974–1982. 12481080 10.1104/pp.010587 PMC166708

[pcaa092-B127] Ozga J.A. , Reinecke D.M. (2003) Hormonal interactions in fruit development. J. Plant Growth Regul. 22: 73–81.

[pcaa092-B128] Patil V. , McDermott H.I., McAllister T., Cummins M., Silva J.C., Mollison E., et al (2019) APETALA2 control of barley internode elongation. Development 146: 170373. 10.1242/dev.170373 PMC6589076 31076487

[pcaa092-B129] Pearce S. , Huttly A.K., Prosser I.M., Li Y.-D., Vaughan S.P., Gallova B., et al (2015) Heterologous expression and transcript analysis of gibberellin biosynthetic genes of grasses reveals novel functionality in the GA3ox family. BMC Plant Biol. 15: 130. 26044828 10.1186/s12870-015-0520-7 PMC4455330

[pcaa092-B130] Pearce S. , Saville R., Vaughan S.P., Chandler P.M., Wilhelm E.P., Sparks C.A., et al (2011) Molecular characterization of *Rht-1* dwarfing genes in hexaploid wheat. Plant Physiol. 157: 1820–1831. 22013218 10.1104/pp.111.183657 PMC3327217

[pcaa092-B131] Peters R.J. (2006) Uncovering the complex metabolic network underlying diterpenoid phytoalexin biosynthesis in rice and other cereal crop plants. Phytochemistry 67: 2307–2317. 16956633 10.1016/j.phytochem.2006.08.009

[pcaa092-B132] Pieterse C.M.J. , Pierik R., van Wees S.C.M. (2014) Different shades of JAZ during plant growth and defense. New Phytol. 204: 261–264. 25236167 10.1111/nph.13029

[pcaa092-B133] Plackett A.R.G. , Powers S.J., Fernandez-Garcia N., Urbanova T., Takebayashi Y., Seo M., et al (2012) Analysis of the developmental roles of the Arabidopsis gibberellin 20-oxidases demonstrates that GA20OX1, -2, and -3 are the dominant paralogs. Plant Cell 24: 941–960. 22427334 10.1105/tpc.111.095109 PMC3336139

[pcaa092-B134] Prisic S. , Peters R.J. (2007) Synergistic substrate inhibition of *ent*-copalyl diphosphate synthase: a potential feed-forward inhibition mechanism limiting gibberellin metabolism. Plant Physiol. 144: 445–454. 17384166 10.1104/pp.106.095208 PMC1913771

[pcaa092-B135] Proebsting W.M. , Hedden P., Lewis M.J., Croker S.J., Proebsting L.N. (1992) Gibberellin concentration and transport in genetic lines of pea—effects of grafting. Plant Physiol. 100: 1354–1360. 16653128 10.1104/pp.100.3.1354 PMC1075789

[pcaa092-B136] Rademacher W. (2016) Chemical regulators of gibberellin status and their application in plant production. Annu. Plant Rev. 49: 359–404.

[pcaa092-B137] Regnault T. , Daviere J.M., Wild M., Sakvarelidze-Achard L., Heintz D., Bergua E.C., et al (2015) The gibberellin precursor GA_12_ acts as a long-distance growth signal in *Arabidopsis*. Nat. Plants 1: 15073. 27250008 10.1038/nplants.2015.73

[pcaa092-B138] Reinecke D.M. , Wickramarathna A.D., Ozga J.A., Kurepin L.V., Jin A.L., Good A.G., et al (2013) Gibberellin 3-oxidase gene expression patterns influence gibberellin biosynthesis, growth, and development in pea. Plant Physiol. 163: 929–945. 23979969 10.1104/pp.113.225987 PMC3793069

[pcaa092-B139] Rieu I. , Ruiz-Rivero O., Fernandez-Garcia N., Griffiths J., Powers S.J., Gong F., et al (2008) The gibberellin biosynthetic genes AtGA20ox1 and AtGA20ox2 act, partially redundantly, to promote growth and development throughout the Arabidopsis life cycle. Plant J. 53: 488–504. 18069939 10.1111/j.1365-313X.2007.03356.x

[pcaa092-B140] Rizza A. , Jones A.M. (2019) The makings of a gradient: spatiotemporal distribution of gibberellins in plant development. Curr. Opin. Plant Biol. 47: 9–15. 30173065 10.1016/j.pbi.2018.08.001 PMC6414749

[pcaa092-B141] Rizza A. , Walia A., Lanquar V., Frommer W.B., Jones A.M. (2017) *In vivo* gibberellin gradients visualized in rapidly elongating tissues. Nat. Plants 3: 803–813. 28970478 10.1038/s41477-017-0021-9

[pcaa092-B142] Rojas M.C. , Hedden P., Gaskin P., Tudzynski B. (2001) The P450-1 gene of *Gibberella fujikuro*i encodes a multifunctional enzyme in gibberellin biosynthesis. Proc. Natl. Acad. Sci. USA 98: 5838–5843. 11320210 10.1073/pnas.091096298 PMC33300

[pcaa092-B143] Ross J.J. , Davidson S.E., Wolbang C.M., Bayly-Stark E., Smith J.J., Reid J.B. (2003) Developmental regulation of the gibberellin pathway in pea shoots. Funct. Plant Biol. 30: 83–89. 32688995 10.1071/FP02108

[pcaa092-B144] Ross J.J. , Miraghazadeh A., Beckett A.H., Quittenden L.J., McAdam E.L. (2016) Interactions between gibberellins and other hormones. Annu. Plant Rev. 2016: 229–252.

[pcaa092-B145] Ross J.J. , Quittenden L.J. (2016) Interactions between brassinosteroids and gibberellins: synthesis or signaling? Plant Cell 28: 829–832. 27006485 10.1105/tpc.15.00917 PMC4863384

[pcaa092-B146] Ross J.J. , Reid J.B. (2010) Evolution of growth-promoting plant hormones. Funct. Plant Biol. 37: 795–805.

[pcaa092-B147] Ross J.J. , Weston D.E., Davidson S.E., Reid J.B. (2011) Plant hormone interactions: how complex are they? Physiol. Plant 141: 299–309. 21214880 10.1111/j.1399-3054.2011.01444.x

[pcaa092-B148] Ruiz-Sola M.�. , Coman D., Beck G., Barja M.V., Colinas M., Graf A., et al (2016) *Arabidopsis* GERANYLGERANYL DIPHOSPHATE SYNTHASE 11 is a hub isozyme required for the production of most photosynthesis-related isoprenoids. New Phytol. 209: 252–264. 26224411 10.1111/nph.13580

[pcaa092-B149] Sakamoto T. , Kobayashi M., Itoh H., Tagiri A., Kayano T., Tanaka H., et al (2001) Expression of a gibberellin 2-oxidase gene around the shoot apex is related to phase transition in rice. Plant Physiol. 125: 1508–1516. 11244129 10.1104/pp.125.3.1508 PMC65628

[pcaa092-B150] Sakamoto T. , Miura K., Itoh H., Tatsumi T., Ueguchi-Tanaka M., Ishiyama K., et al (2004) An overview of gibberellin metabolism enzyme genes and their related mutants in rice. Plant Physiol. 134: 1642–1653. 15075394 10.1104/pp.103.033696 PMC419838

[pcaa092-B151] Schneider G. , Jensen E., Spray C.R., Phinney B.O. (1992) Hydrolysis and reconjugation of gibberellin A_20_ glucosyl ester by seedlings of *Zea mays* L. Proc. Natl. Acad. Sci. USA 89: 8045–8048. 1518829 10.1073/pnas.89.17.8045 PMC49852

[pcaa092-B152] Schrager-Lavelle A. , Gath N.N., Devisetty U.K., Carrera E., Lopez-Diaz I., Blazquez M.A., et al (2019) The role of a class III gibberellin 2-oxidase in tomato internode elongation. Plant J. 97: 603–615. 30394600 10.1111/tpj.14145

[pcaa092-B153] Shimane M. , Ueno Y., Morisaki K., Oogami S., Natsume M., Hayashi K., et al (2014) Molecular evolution of the substrate specificity of *ent*-kaurene synthases to adapt to gibberellin biosynthesis in land plants. Biochem. J. 462: 539–546. 24983886 10.1042/BJ20140134

[pcaa092-B154] Silverstone A.L. , Chang C.W., Krol E., Sun T.P. (1997) Developmental regulation of the gibberellin biosynthetic gene GA1 in *Arabidopsis thaliana*. Plant J. 12: 9–19. 9263448 10.1046/j.1365-313x.1997.12010009.x

[pcaa092-B155] Smith V.A. (1992) Gibberellin A_1_ biosynthesis in *Pisum sativum* L. 2. Biological and biochemical consequences of the LE mutation. Plant Physiol. 99: 372–377. 16668893 10.1104/pp.99.2.372 PMC1080470

[pcaa092-B156] Sponsel V.M. (2016) Signal achievements in gibberellin research: the second half-century. Annu. Plant Rev. 49: 1–36.

[pcaa092-B157] Stavang J.A. , Gallego-Bartolome J., Gomez M.D., Yoshida S., Asami T., Olsen J.E., et al (2009) Hormonal regulation of temperature-induced growth in *Arabidopsis*. Plant J. 60: 589–601. 19686536 10.1111/j.1365-313X.2009.03983.x

[pcaa092-B158] Sun H. , Pang B.Y., Yan J., Wang T., Wang L.N., Chen C.H., et al (2018) Comprehensive analysis of cucumber gibberellin oxidase family genes and functional characterization of CsGA20ox1 in root development in *Arabidopsis*. Int. J. Mol. Sci. 19: 3135. 10.3390/ijms19103135 PMC6213227 30322023

[pcaa092-B159] Sun T.P. (2011) The molecular mechanism and evolution of the GA-GID1-DELLA signaling module in plants. Curr. Biol. 21: R338–R345. 21549956 10.1016/j.cub.2011.02.036

[pcaa092-B160] Sun T.P. , Kamiya Y. (1994) The Arabidopsis GA1 locus encodes the cyclase *ent*-kaurene synthetase A of gibberellin biosynthesis. Plant Cell 6: 1509–1518. 7994182 10.1105/tpc.6.10.1509 PMC160538

[pcaa092-B161] Sun T.P. , Kamiya Y. (1997) Regulation and cellular localization of *ent*-kaurene synthesis. Physiol. Plant. 101: 701–708.

[pcaa092-B162] Takahashi N. , Kitamura H., Kawarada A., Seta Y., Takai M., Tamura S., et al (1955) Biochemical studies on ‘‘Bakanae” fungus. Part XXXIV. Isolation of gibberellins and their properties. Bull. Agric. Chem. Soc. Jpn. 19: 267–277.

[pcaa092-B163] Takehara S. , Sakuraba S., Mikama B., Yoshida H., Yoshimura H., Itoh A., et al (2020) A common allosteric mechanism regulates homeostatic inactivation of auxin and gibberellin. Nat. Commun. 11: 2143. 32358569 10.1038/s41467-020-16068-0 PMC7195466

[pcaa092-B164] Tanaka J. , Yano K., Aya K., Hirano K., Takehara S., Koketsu E., et al (2014) Antheridiogen determines sex in ferns via a spatiotemporally split gibberellin synthesis pathway. Science 346: 469–473. 25342803 10.1126/science.1259923

[pcaa092-B165] Tatsukami Y. , Ueda M. (2016) Rhizobial gibberellin negatively regulates host nodule number. Sci. Rep. 6: 27998. 27307029 10.1038/srep27998 PMC4910070

[pcaa092-B166] Thomas S.G. , Blazquez M.A., Alabadi D. (2016) DELLA proteins: master regulators of gibberellin-responsive growth and development. Annu. Plant Rev. 49: 189–228.

[pcaa092-B167] Thomas S.G. , Phillips A.L., Hedden P. (1999) Molecular cloning and functional expression of gibberellin 2-oxidases, multifunctional enzymes involved in gibberellin deactivation. Proc. Natl. Acad. Sci. USA 96: 4698–4703. 10200325 10.1073/pnas.96.8.4698 PMC16395

[pcaa092-B168] Tudzynski B. (2014) Nitrogen regulation of fungal secondary metabolism in fungi. Front. Microbiol. 5: 565. 25506342 10.3389/fmicb.2014.00656 PMC4246892

[pcaa092-B169] Tudzynski B. , Hedden P., Carrera E., Gaskin P. (2001) The P450-4 gene of *Gibberella fujikuroi* encodes *ent*-kaurene oxidase in the gibberellin biosynthesis pathway. Appl. Environ. Microbiol. 67: 3514–3522. 11472927 10.1128/AEM.67.8.3514-3522.2001 PMC93051

[pcaa092-B170] Tudzynski B. , Mihlan M., Rojas M.C., Linnemannstons P., Gaskin P., Hedden P. (2003) Characterization of the final two genes of the gibberellin biosynthesis gene cluster of *Gibberella fujikuroi* des and P450-3 encode GA_4_ desaturase and the 13-hydroxylase, respectively. J. Biol. Chem. 278: 28635–28643. 12750377 10.1074/jbc.M301927200

[pcaa092-B171] Tudzynski B. , Rojas M.C., Gaskin P., Hedden P. (2002) The gibberellin 20-oxidase of *Gibberella fujikuroi* is a multifunctional monooxygenase. J. Biol. Chem. 277: 21246–21253. 11943776 10.1074/jbc.M201651200

[pcaa092-B172] Ueguchi-Tanaka M. , Ashikari M., Nakajima M., Itoh H., Katoh E., Kobayashi M., et al (2005) *GIBBERELLIN INSENSITIVE DWARF1* encodes a soluble receptor for gibberellin. Nature 437: 693–698. 16193045 10.1038/nature04028

[pcaa092-B173] van Schie C.C.N. , Ament K., Schmidt A., Lange T., Haring M.A., Schuurink R.C. (2007) Geranyl diphosphate synthase is required for biosynthesis of gibberellins. Plant J. 52: 752–762. 17877699 10.1111/j.1365-313X.2007.03273.x

[pcaa092-B174] Varbanova M. , Yamaguchi S., Yang Y., McKelvey K., Hanada A., Borochov R., et al (2007) Methylation of gibberellins by Arabidopsis GAMT1 and GAMT2. Plant Cell 19: 32–45. 17220201 10.1105/tpc.106.044602 PMC1820973

[pcaa092-B175] Wang Q. , Hillwig M.L., Wu Y.S., Peters R.J. (2012) CYP701A8: a rice ent-kaurene oxidase paralog diverted to more specialized diterpenoid metabolism. Plant Physiol. 158: 1418–1425. 22247270 10.1104/pp.111.187518 PMC3291257

[pcaa092-B176] Ward D.A. , MacMillan J., Gong F., Phillips A.L., Hedden P. (2010) Gibberellin 3-oxidases in developing embryos of the southern wild cucumber, *Marah macrocarpus*. Phytochemistry 71: 2010–2018. 20965527 10.1016/j.phytochem.2010.09.015

[pcaa092-B177] Ward J.L. , Gaskin P., Brown R.G.S., Jackson G.S., Hedden P., Phillips A.L., et al (2002) Probing the mechanism of loss of carbon-20 in gibberellin biosynthesis. Synthesis of gibberellin 3α,20-hemiacetal and 19,20-lactol analogues and their metabolism by a recombinant GA 20-oxidase. J. Chem. Soc. Perkin Trans. 1 1: 232–241.

[pcaa092-B178] Ward J.L. , Jackson G.J., Beale M.H., Gaskin P., Hedden P., Mander L.N., et al (1997) Stereochemistry of the oxidation of gibberellin 20-alcohols. Chem. Commun. 13–14.

[pcaa092-B179] Weiss D. , Halevy A.H. (1989) Stamens and gibberellin in the regulation of corolla pigmentation and growth in *Petunia hybrida*. Planta 179: 89–96. 24201426 10.1007/BF00395775

[pcaa092-B180] Wexler S. , Schayek H., Rajendar K., Tal I., Shani E., Meroz Y., et al (2019) Characterizing gibberellin flow in planta using photocaged gibberellins. Chem. Sci. 10: 1500–1505. 30809367 10.1039/c8sc04528c PMC6354844

[pcaa092-B181] Wiemann P. , Sieber C.M.K., Von Bargen K.W., Studt L., Niehaus E.M., Espino J.J., et al (2013) Deciphering the cryptic genome: genome-wide analyses of the rice pathogen *Fusarium fujikuroi* reveal complex regulation of secondary metabolism and novel metabolites. PLoS Pathog. 9: e1003475. 23825955 10.1371/journal.ppat.1003475 PMC3694855

[pcaa092-B182] Wu Y.S. , Zhou K., Toyomasu T., Sugawara C., Oku M., Abe S., et al (2012) Functional characterization of wheat copalyl diphosphate synthases sheds light on the early evolution of labdane-related diterpenoid metabolism in the cereals. Phytochemistry 84: 40–46. 23009878 10.1016/j.phytochem.2012.08.022 PMC3483432

[pcaa092-B183] Xing S.F. , Qin G.J., Shi Y., Ma Z.Q., Chen Z.L., Gu H.Y., et al (2007) GAMT2 encodes a methyltransferase of gibberellic acid that is involved in seed maturation and germination in *Arabidopsis*. J. Integr. Plant Biol. 49: 368–381.

[pcaa092-B184] Xiong W. , Ye T.T., Yao X., Liu X., Ma S., Chen X., et al (2018) The dioxygenase GIM2 functions in seed germination by altering gibberellin production in *Arabidopsis*. J. Integr. Plant Biol. 60: 276–291. 29205921 10.1111/jipb.12619

[pcaa092-B185] Xu M.M. , Wilderman P.R., Morrone D., Xu J.J., Roy A., Margis-Pinheiro M., et al (2007) Functional characterization of the rice kaurene synthase-like gene family. Phytochemistry 68: 312–326. 17141283 10.1016/j.phytochem.2006.10.016

[pcaa092-B186] Yabuta T. , Sumiki T. (1938) Communication to the editor. J. Agric. Chem. Soc. Jpn. 14: 1526.

[pcaa092-B187] Yamaguchi N. , Winter C.M., Wu M.F., Kanno Y., Yamaguchi A., Seo M., et al (2014) Gibberellin acts positively then negatively to control onset of flower formation in *Arabidopsis*. Science 344: 638–641. 24812402 10.1126/science.1250498

[pcaa092-B188] Yamaguchi S. , Saito T., Abe H., Yamane H., Murofushi N., Kamiya Y. (1996) Molecular cloning and characterization of a cDNA encoding the gibberellin biosynthetic enzyme *ent*-kaurene synthase B from pumpkin (*Cucurbita maxima* L.). Plant J. 10: 203–213. 8771778 10.1046/j.1365-313x.1996.10020203.x

[pcaa092-B189] Yamaguchi S. , Sun T., Kawaide H., Kamiya Y. (1998) The *GA2* locus of *Arabidopsis thaliana* encodes *ent*-kaurene synthase of gibberellin biosynthesis. Plant Physiol. 116: 1271–1278. 9536043 10.1104/pp.116.4.1271 PMC35033

[pcaa092-B190] Yumane H. , Satoh Y., Nohara K., Nakayama M., Murofushi N., Takahashi N., et al (1988) The methyl ester of a new gibberellin, GA_73_—the principal antheridiogen in *Lygodium japonicum*. Tetrahedron Lett. 29: 3959–3962.

[pcaa092-B191] Yoshida H. , Hirano K., Sato T., Mitsuda N., Nomoto M., Maeo K., et al (2014) DELLA protein functions as a transcriptional activator through the DNA binding of the INDETERMINATE DOMAIN family proteins. Proc. Natl. Acad. Sci. USA 111: 7861–7866. 24821766 10.1073/pnas.1321669111 PMC4040565

[pcaa092-B192] Zhang H. , Li M., He D.L., Wang K., Yang P.F. (2020) Mutations on *ent*-kaurene oxidase 1 encoding gene attenuate its enzyme activity of catalyzing the reaction from *ent*-kaurene to *ent*-kaurenoic acid and lead to delayed germination in rice. PLoS Genet. 16: e1008562. 31923187 10.1371/journal.pgen.1008562 PMC6977763

[pcaa092-B193] Zhang Y.F. , Su P., Wu X.Y., Zhou J.W., Zhao Y.J., Hu T.Y., et al (2019) The gibberellin 13-oxidase that specifically converts gibberellin A_9_ to A_20_ in *Tripterygium wilfordii* is a 2-oxoglutarate-dependent dioxygenase. Planta 250: 1613–1620. 31388830 10.1007/s00425-019-03240-0

[pcaa092-B194] Zhang Y.Y. , Zhang B.C., Yan D.W., Dong W.X., Yang W.B., Li Q., et al (2011) Two *Arabidopsis* cytochrome P450 monooxygenases, CYP714A1 and CYP714A2, function redundantly in plant development through gibberellin deactivation. Plant J. 67: 342–353. 21457373 10.1111/j.1365-313X.2011.04596.x

[pcaa092-B195] Zhou F. , Wang C.Y., Gutensohn M., Jiang L., Zhang P., Zhang D.B., et al (2017) A recruiting protein of geranylgeranyl diphosphate synthase controls metabolic flux toward chlorophyll biosynthesis in rice. Proc. Natl. Acad. Sci. USA 114: 6866–6871. 28607067 10.1073/pnas.1705689114 PMC5495272

[pcaa092-B196] Zhou K. , Peters R.J. (2009) Investigating the conservation pattern of a putative second terpene synthase divalent metal binding motif in plants. Phytochemistry 70: 366–369. 19201430 10.1016/j.phytochem.2008.12.022 PMC2682547

[pcaa092-B197] Zhou K. , Xu M.M., Tiernan M., Xie Q., Toyomasu T., Sugawara C., et al (2012) Functional characterization of wheat *ent*-kaurene(-like) synthases indicates continuing evolution of labdane-related diterpenoid metabolism in the cereals. Phytochemistry 84: 47–55. 23009879 10.1016/j.phytochem.2012.08.021 PMC3483413

[pcaa092-B198] Zhu Y.Y. , Nomura T., Xu Y.H., Zhang Y.Y., Peng Y., Mao B.Z., et al (2006) *ELONGATED UPPERMOST INTERNODE* encodes a cytochrome P450 monooxygenase that epoxidizes gibberellins in a novel deactivation reaction in rice. Plant Cell 18: 442–456. 16399803 10.1105/tpc.105.038455 PMC1356550

[pcaa092-B199] Zi J.C. , Mafu S., Peters R.J. (2014) To gibberellins and beyond! Surveying the evolution of (di)terpenoid metabolism. Annu. Rev. Plant Biol. 65: 259–286. 24471837 10.1146/annurev-arplant-050213-035705 PMC4118669

